# Effect of artificial gravity on neurocognitive performance during head-down tilt bedrest

**DOI:** 10.1038/s41526-024-00405-4

**Published:** 2024-06-05

**Authors:** Borbála Tölgyesi, Anna Altbäcker, Irén Barkaszi, Tim Stuckenschneider, Leonard Braunsmann, Endre Takács, Bea Ehmann, László Balázs, Vera Abeln

**Affiliations:** 1 Institute of Cognitive Neuroscience and Psychology, HUN-REN Research Centre for Natural Sciences, Budapest, Hungary; 2 Interaction and Immersion Hub, Innovation Center, Moholy-Nagy University of Art and Design, Budapest, Hungary; 3 Institute of Movement and Neurosciences, Centre for Health and Integrative Physiology in Space (CHIPS), German Sport University Cologne, Cologne, Germany; 4 Geriatric Medicine, Department for Health, Services Research, School of Medicine and Health Sciences, Carl von Ossietzky University, Oldenburg, Germany

**Keywords:** Risk factors, Neuroscience

## Abstract

This study evaluated the acute and chronic effects of intermittent and continuous Artificial Gravity (AG) on cognitive performance during 60 days of Head-down tilt bedrest (HDTBR), a well-established ground-based spaceflight analogue method. Participants were randomly assigned to three groups: intermittent AG, continuous AG, and HDTBR control group without AG exposure. Task performance and electrophysiological measures of attention and working memory were investigated during Simple and Complex tasks in the Visual and the Auditory modality. Compared to baseline, faster reaction time and better accuracy was present during HDTBR regarding the Complex tasks, however, the practice effect was diminished in the three HDTBR groups compared to an ambulatory control group. Brain potentials showed a modality-specific decrease, as P3a was decreased only in the Auditory, while P3b decreased in the Visual modality. No evidence for acute or chronic AG-related cognitive impairments during HDTBR was found.

## Introduction

Extreme environmental conditions such as microgravity can be physiologically and cognitively challenging for humans. Since sustained high level of cognitive performance in astronauts is a prerequisite for successful space missions, it is important to assess the adverse effects of prolonged exposure to space-related stressors (e.g. microgravity, cosmic radiation, elevated CO2 levels, altered light exposure, increased ambient noise) and challenges related to the spaceflight environment (e.g. high workload, isolation, and confinement) (for a review see^1^). Astronauts experience physiological deconditioning during spaceflights^2^, including loss of muscle mass and strength, bone density, sensory-motor deconditioning, altered central blood volume regulation, and headward fluid shift^3^. Growing neuroimaging evidence shows that brain structure is affected by spaceflight including the upward shift of the brain, increased total ventricular volume^4^ along with decreased gray matter and white matter volume^5,6^. Despite the clear impact of spaceflight on the central nervous system, results regarding spaceflight-related cognitive symptoms are mixed: while some studies report cognitive deficits, such as disturbed visuospatial performance^7–9^, others failed to demonstrate significant changes in cognitive performance (for reviews see^1,10,11^). The reason behind inconsistent results includes low sample sizes and relatedly low statistical power, high variation in cognitive test batteries and targeted cognitive domains, and lack of more controllable settings, such as ground-based analogs^1,12^).

Head-down tilt bedrest (HDTBR) presents a well-established ground-based space analog model for microgravity-induced physiological alterations including cardiovascular changes^3,13^, cephalic fluid shift^14^, upward brain shift, structural brain changes^15,16^. Even though it is suggested that HDTBR has a similar impact on cognitive functions as spaceflight, results regarding HDTBR on cognitive performance are also far from being conclusive (for reviews see^1,17^). HDTBR also presents a great opportunity to test the effectiveness of countermeasures against microgravity-induced physiological changes under terrestrial conditions, such as short-arm centrifugation. By generating Artificial Gravity (AG), short-arm centrifugation simulates the gravitational forces on Earth creating a quasi-equivalent load on the physiological systems along with presenting an orthostatic challenge that the human body experiences under terrestrial conditions^18^. AG also has the potential to counteract headward fluid shift and enabling cerebrospinal fluid outflow^19^. However, centrifugation can cause pre-syncopal symptoms and motion sickness which affect behavioral performance^20^. Thus, it is important to determine the optimal short-arm centrifugation protocol, which is effective whilst remaining tolerable^21,22^. The effects of short-arm centrifugation on cognitive performance during long-term HDTBR, and obviously during space missions is an open question. Therefore, it would be beneficial to evaluate the acute as well as long-term effects of short-arm centrifugation during long-term HDTBR on cognitive processes by combining behavioral measures of cognitive performance with simultaneous recording of brain functions.

Electroencephalography (EEG) presents a non-invasive method to monitor brain functions and has been used in recent experiments on the International Space Station^9,23^, as its technical and operational requirements allow the use in space in contrast to (f)MRI systems. Cognitive processing is well reflected by event-related potentials (ERPs) of EEG, especially late positive components P3a and P3b, representing automatic and controlled attentional processes^24,25^. The P3a is a component with fronto-central scalp distribution, elicited by rare task-irrelevant deviant/novel stimuli. Unlike P3a, the P3b component is typically elicited by Target stimuli and has a centro-parietal scalp distribution. A diminished amplitude of either component may indicate cognitive decline due to stressors, such as sleep loss^26,27^, hypoxia^28,29^, and mental fatigue^30^.

The current study aimed at investigating how the HDTBR induced well-known cephalic fluid shift and upward brain shift affect cognitive abilities by measuring behavioral performance along with simultaneous recording of electrophysiological responses. To identify potential modality-specific or generalized cognitive deterioration related to HDTBR itself/ and or AG exposure, participants performed four cognitive tasks, two in the Auditory and two in the Visual modality. Two levels of task complexity (Simple and Complex) were used in each modality to specify the affected neurocognitive domains responsible for any significant performance decrement. Performance deterioration in Simple tasks indicates reduced psychomotor speed, while impaired performance in Complex tasks indicates reduced working memory capacity. Additionally, Complex tasks used in this study were designed to enable the investigation of brain electrical activity as they include stimuli (i.e. rare Irrelevant and Target stimuli) that typically elicit P3a and P3b components, the ERP correlates of attention. The applied tasks also help to elucidate if impairments are specific to the Visual or the Auditory domain.

We hypothesized that behavioral measures of task performance (reaction time, Accuracy) as well as the amplitudes of P3a and P3b ERP components would be altered by 60 days of 6° HDTBR exposure and that the impact of HDTBR on cognitive task performance is affected by task complexity. In addition, we aimed to compare the cognitive effects of HDTBR itself and the counterbalancing effects of short-arm centrifugation during HDTBR. We hypothesized that short-arm centrifugation would attenuate the negative impact of HDTBR on cognitive functions, and predicted cognitive deterioration of prolonged bedrest would be less pronounced in groups receiving AG as a countermeasure. Furthermore, we also aimed to evaluate whether intermittent and continuous centrifugation protocols affect the cognitive system differently. Finally, we aimed to separate the acute and chronic effects of AG. We expected that, acutely, the visual system would be more disturbed by centrifugation than the auditory system, especially for the continuous protocol.

## Methods

### Design

Our study was conducted in 2019 at the :envihab facility of the German Aerospace Center (DLR, Germany, Cologne). This study was part of the Artificial Gravity Bed Rest - European Space Agency (AGBRESA) project, which was designed to compare the counterbalancing effects of intermittent and continuous Artificial Gravity on the microgravity-induced physiological changes to 60 days of HDTBR to identify an appropriate countermeasure protocol for future space missions^31^. The AGBRESA project consisted of two campaigns. Each campaign included fourteen days of baseline data collection (BDC-14 through BDC-1), 60 days of HDTBR (HDT1 through HDT60), and fourteen days of recovery (R + 0 through R + 13). The primary objective of the AGBRESA bedrest study was to compare the tolerability and protective effects of two different centrifugation protocols against HDTBR. Participants were randomly assigned to three different groups, which either received intermittent (iAG) or continuous (cAG) centrifugation or acted as a control group without AG exposure (ctrlAG). The iAG group underwent 60 days of 6° HDTBR with supine centrifugation for 6 bouts of 5 minutes per day which were separated by 3 minutes of rest. The cAG group also underwent 60 days of 6° HDTBR with 30 minutes of uninterrupted supine centrifugation per day (for more details, see the section HDTBR and Centrifugation Protocol below). The ctrlAG group underwent 60 days of 6° HDTBR without centrifugation.

**Fig. 1:**
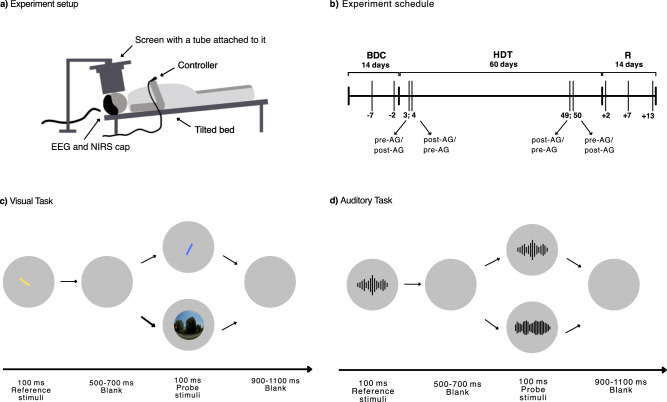
Schematic figure of the experiment setup, experiment timeline, and tasks. **a** Experiment setup. Participants performed the experimental tasks in a lying position, looking at a computer screen via a cylindrical tube and responses were given using a controller. They wore an EEG cap linked with NIRS optodes throughout performing the experimental tasks. **b** Schematic figure of the experiment timeline and the HDTBR Phases regarding the HDTBR groups. AMBCtrl group had the same timeline without undergoing HDTBR and AG. **c** Stimulus presentation in the Visual tasks. Participants had to indicate whether two successively presented lines (Reference and Probe stimuli) had the same orientation or not. Twenty percent of the Probe stimuli were replaced by a task-irrelevant picture (Irrelevant stimulus). **d** Stimulus presentation in the Auditory tasks. Participants had to indicate whether two successively presented sound beeps (Reference and Probe stimuli) had the same pitch or not. Twenty percent of the Probe stimuli were replaced by task-irrelevant environmental noises (Irrelevant stimulus).

Our study design involved nine data collection points, two during BDC before the bedrest period, (BDC-8, BDC-4), four during bedrest (HDT3, HDT4, HDT49, HDT50), and three after bedrest (R + 2, R + 7, R + 13). To evaluate the acute and chronic effects of AG, both early (HDT3, HDT4) and late (HDT49, HDT50) bedrest measurements consisted of two sessions conducted during consecutive days, with one session scheduled directly after centrifugation (Acute effects), and the other one before centrifugation (Chronic effects). The order of pre- and post-centrifugation measurements was counterbalanced between the two campaigns, therefore, pre-centrifugation sessions were scheduled at HDT3 and HDT50, and post-centrifugation sessions were scheduled at HDT4 and HDT49 for the first campaign, while pre-centrifugation sessions were scheduled at HDT4 and HDT49 and post-centrifugation sessions were scheduled HDT3 and HDT50 for the second campaign. Task familiarization was performed on BDC-13 and BDC-10. For a schematic depiction of the study design see Fig. 1a-b.

Previous results showed that visual cognitive tasks including the ones used in this study may be sensitive to the practice effect (Takács et al.^9^). Therefore, an ambulatory control group (AMBCtrl) was further included to the study design, which did not receive AG exposure and did not have to stay in bed for 60 days but otherwise followed a similar test protocol and schedule as the three HDTBR experimental groups. AMBCtrl data was collected at the German Sport University Cologne, and they only had to stay there during their measurements which were taken in a horizontal supine position. Their data was used to compare the practice effect related to repeated task administration with the HDTBR groups.

### Subjects

Twenty-four participants were recruited for the HDTBR study by the DLR. Inclusion and exclusion criteria are detailed elsewhere^22,32^. The study was conducted during two campaigns with 12 subjects randomly assigned to the three experimental groups in each campaign (iAG, cAG, ctrlAG; n = 8 in each group). Out of twenty-four subjects, two participants were excluded from the analysis, one from the iAG group due to low behavioral performance (in both Complex tasks Accuracy was worse than 60% during at least 3 sessions) and one from the ctrlAG group due to low EEG quality (number of sweeps was below 20 in each condition during at least an entire session). Thus, the final HDTBR sample comprised twenty-two participants, seven in the iAG, (age M = 32, SD = 10.34, 2 females), eight in the cAG (age M = 31.87, SD = 9.75, 3 females), and seven in ctrlAG, (age M = 35.71, SD = 7.20, 2 females). For the AMBCtrl, the same inclusion and exclusion criteria were applied. Additionally, subjects in the AMBCtrl group were matched with those in the three HDTBR groups based on age and level of education. Ten individuals were recruited through leaflets and a research newsletter. One participant was excluded from the analysis due to low behavioral performance (showing 0% Accuracy during one session), thus, the final sample comprised nine participants (age M = 32.11 SD = 9.66, 4 female).

All subjects received monetary compensation for participating in the study. Before taking part in the study, all participants gave written informed consent to the experimental procedures. The AGBRESA project was approved by the Ethics committee of the Northern Rhine Medical Association (Ärztekammer Nordrhein, application No. 2018143) in Düsseldorf, Germany, as well as the Federal Office for Radiation Protection (Bundesamt für Strahlenschutz, application No. 22464/2018–074-R-G), while the ethical approval for the AMBCtrl was given by the Ethics committee of the German Sport University Cologne (No. 173/2018). The study was registered at the German Clinical Trials Register (DRKS00015677). The experimental protocol complied with all guidelines stated in the Declaration of Helsinki.

### HDTBR and Centrifugation Protocol

Participants could move freely within the ward during the BDC and R phases, while all activities were performed during a strict 6° HDTBR position for 24 *h*/day during the entire HDT Phase. The applied HDTBR protocol followed the International Guidelines for Standardization of bedrest studies in the spaceflight context^33^. During HDTBR, participants of the iAG and cAG groups were daily exposed to +1 *Gz* at the center of mass and approximately +2 *Gz* at foot level using the :envihab short-arm human centrifuge. Participants were instructed to stay in a supine position during centrifugation, gaze fixed on a point, avoid head movements and only contract the leg muscles to avoid pre-syncope symptoms. All subjects underwent two centrifuge familiarization sessions before bedrest (BDC-14 and BDC-4). Further details of the AGBRESA HDTBR and centrifugation protocol are available at^22,31,34^.

### Stimuli and procedure

#### Cognitive tasks

All participants completed the four following tasks along with concomitant EEG measurement. These tasks were designed to identify modality-specific or generalized cognitive deterioration and to specify the neurocognitive domains responsible for any significant performance decrement in response to HDTBR and/or AG exposure. Out of the four tasks, two were Visual and two were Auditory tasks, both with two levels of complexity. Visual stimuli were presented on a monitor seen through a tunnel which was intended to shield external visual stimuli. The monitor was placed on a stand above the bed and centered on the line of gaze at 25 *cm* from the eyes. The tunnel had a diameter of 22 *cm*. Auditory stimuli were presented through noise-reduction headphones (Bose QuietComfort20) and a gamepad (Microsoft Xbox One Controller) was used as the response device.

One session took approximately forty minutes, using the following structure: ten minutes for EEG cap preparation, one minute of open-eye and one minute of closed-eye EEG baseline measurement, three blocks of cognitive tasks, one minute of open-eye measurement, and three blocks of tasks. The blocks were approximately 3.5 minutes long and were separated by short breaks of 20 seconds. Participants were randomly assigned to two counterbalanced task orders (order A: Auditory Simple, Auditory Complex, Visual Complex, Visual Simple, Visual Complex, Auditory Complex; order B: Visual Complex, Visual Simple, Auditory Complex, Auditory Complex, Auditory Simple, Visual Complex).

The Visual Complex task is a match-to-sample task which is a classic probe of working memory frequently employed in human and primate research^35^. This version of the task has been previously used in a spaceflight experiment (Takács et al.^9^). Each trial started with the presentation of the Reference stimulus (S1). The S1 featured a yellow line in the center of fixation oriented at 0°, 30°, 60°, 90°, 120° or 150° from horizontal for 100 milliseconds (*ms*) on a gray background. After a 500–700 *ms* long interstimulus interval (ISI), S1 was followed by a Probe stimulus (S2, Target stimulus, 100 *ms*), a blue line, which was always different from S1 in location and could either have the same orientation as S1 or could be different from it by 30° or 60° (Fig. 1c). Subjects had to indicate whether S1 and S2 had the same orientation by pressing a YES or a NO button. In 20% of the trials, S2 was replaced by various pictures (Irrelevant stimuli). Irrelevant stimuli elicit the orientation response that manifests in specific event-related potentials (P3a) on frontal-central areas of the scalp^24,36^. Subjects had to ignore these stimuli and withhold any responses. The intertrial interval (ITI) was 1600–1800 *ms*. The screen resolution was 1920 × 1080 pixels, while the dimensions of the presented stimuli were 83 × 7 pixels for S1 and S2 and 200 × 200 pixels for Irrelevant stimuli.

The Visual Simple task is a simple reaction time task that uses the same set of stimuli as the Visual Complex task. This task is suitable for measuring attention and psychomotor speed. However, in this task subjects had to press the YES button whenever S2 appeared regardless of its orientation relative to the S1. Still, they had to ignore Irrelevant stimuli. S1 was displayed for 100 *ms* followed by a 500–700 *ms* ISI. Then, an S2 or an Irrelevant stimulus was presented (in 20% of the trials) for 100 *ms*. The ITI was 1600–1800 *ms*.

The Auditory Complex match-to-sample task is a close replica of the Visual Complex task which requires working memory and attention. The Reference stimuli (S1) were tones with a pitch out of six possible frequency values. The Probe stimuli (S2, Target stimuli) were tones with either the same or a different pitch. The pitch difference was one or two intervals from S1. In 20% of the trials, S2 is replaced by various environmental noises (Irrelevant stimuli, e.g. bell, phone, door slamming). Subjects had to indicate by pressing the YES or NO button, if S1 and S2 were the same or different pitch and ignore Irrelevant stimuli. The participants were instructed to look at a fixation point on the screen during the task (40 × 40 pixels). The S1 tone lasted for 100 *ms*, followed by a 500–700 *ms* ISI and either by a 100 *ms* long S2 or an Irrelevant stimulus. The ITI was 1600–1800 *ms* (Fig. 1d).

Stimuli in the Auditory Simple task were the same as in the Auditory Complex task. Subjects had to press the YES button whenever S2 was presented to them (80%) and ignore Irrelevant stimuli (20%). Similarly to the Visual Simple task, this was also an easy, non-demanding vigilance task for measuring attention and psychomotor speed. The S1 was present for 100 *ms* followed by a 500–700 *ms* ISI. S2 or Irrelevant stimuli were also present for 100 *ms*, the ITI was 1600–1800 *ms*.

### EEG recording and analysis

During all sessions, EEG was recorded with a sampling frequency of 500 *Hz* using the BrainAmp and 32 active electrodes mounted in flexible BrainCaps (Brain Products GmbH, Gilching, Germany) suitable for individual head sizes (holding also the NIRS optodes). 30 scalp electrodes were placed using the extended 10–20 system: FPz, F7, F3, Fz, F4, F8, FT9, FC1, FC2, FT10, T7, C3, Cz, C4, T8, TP9, CP5, CP1, CPz, CP2, CP6, TP10, P7, P3, Pz, P4, P8, O1, Oz, O2^37^. Two electrodes were attached to both earlobes for re-reference purposes, but they were not used due to noise. Two additional scalp electrodes functioned as a ground (AFz) and as a reference electrode (FCz). Results for NIRS are reported elsewhere.

EEG was analyzed with the EEGLAB toolbox^38^. Data was band-pass filtered off-line 0.5–40 *Hz* (high-pass: Kaiser window, transition bandwidth: 0.5 *Hz*, passband deviation: 0.001 *Hz*; low-pass: Kaiser window, transition bandwidth: 10 *Hz*, passband deviation: 0.001 *Hz*). After the removal of gross artifacts (noisy time segments and channels) by visual inspection, data was re-referenced to representational state transfer (REST) reference^39^ and was subject to extended independent component analysis (ICA) transformation^37,40^. Eye and muscle-related ICA components were identified by inspecting the component scalp map, time course, and ERP-image (visualization of event-related signal variations across single trials) and rejected from further analysis. As a next step, manually deleted channels (M/session/person = 0.6, SD = 0.84) were interpolated using the spherical method with the pop_interp() function in EEGLAB. Data was epoched to −100 to 900 *ms* segments to stimulus events, and baseline corrected (−100 to 0 *ms*). Only trials with correct responses were analyzed. Epochs with a signal range exceeding 70 *μV* on frontal channels and 100 *μV* on non-frontal channels were discarded from the analyses (remaining number of sweeps/session/person for Target stimuli: M = 173.87, SD = 14.93, remaining number of sweeps/session/person for Novel stimuli: M = 45.64, SD = 3.87). Grand means were computed from the individual averages for each stimulus type in each task.

The ERP analysis was focused on the P3a ERP component elicited by Irrelevant stimuli, and the P3b component elicited by Target stimuli. Given its design, Simple tasks lacked typical Target stimuli which elicit P3b and had a limited number of Irrelevant stimuli, therefore, P3a and P3b analyses were conducted for the Visual and the Auditory Complex tasks only. P3a peak latencies were computed on the grand-mean ERP waveforms averaged across subjects and sessions separately for the Visual and Auditory Complex tasks as a positive peak at the average of FC1, FC2, Fz, and Cz electrodes within 220–420 *ms* for the Auditory Complex task and within 280–480 *ms* for the Visual Complex task after the onset of the Irrelevant stimuli. P3a peak latency was 366 *ms* for the Visual, and 324 *ms* for the Auditory Complex task. Mean P3a amplitude was evaluated in a 100 *ms* time window centered at peak latency. P3b peak latencies were identified in the grand-mean ERP waveforms averaged across subjects and sessions separately for the Visual and Auditory Complex tasks as a positive peak at the average of CP1, CP2, CPz, and Pz electrodes within 250–550 *ms* for the Auditory Complex task and within 280–580 *ms* for the Visual Complex task, after the onset of the Probe stimuli. P3b peak latency was 502 *ms* for the Visual, and 510 *ms* for the Auditory Complex task. Mean P3b amplitude was evaluated in a 100 *ms* time window centered at peak latency.

### Questionnaires

Subjective sleepiness was assessed by the Karolinska Sleepiness Scale (KSS^41^), which is suggested to be related to EEG and behavioral parameters of sleepiness, indicating a high validity in measuring sleepiness^42^. KSS was assessed at the beginning of each session in all groups. Results regarding KSS are detailed in the Supplementary Notes, Supplementary Tables 8–10, and Supplementary Tables 21–23.

### Data analysis

For behavioral measures, reaction time (RT) and Accuracy were investigated during all four experimental tasks. To eliminate reactions reflecting fast guesses, only correct responses with a duration greater than 200 *ms* after Probe stimuli offset were included in the analyses. Median RT was calculated for each session, stimulus type, and subject for all tasks. Accuracy was calculated as the percentage of correct button presses for Probe stimuli (press YES/NO) and Novel stimuli (no button pressing) in all tasks. As for the ERP measures, P3a and P3b amplitudes were analyzed separately.

All statistical analyses were conducted using Statistica 13.5 (Dell Inc., 2016). For the investigation of the HDTBR experimental groups, RT and Accuracy were both analyzed using Time (1:9) x Complexity (Simple, Complex) x Group (iAG, cAG, ctrlAG) repeated measures ANOVAs (rANOVA). The two modalities (Visual and Auditory) were analyzed separately. P3a and P3b amplitudes were analyzed using a Time (1:9) x Group (iAG, cAG, ctrlAG) rANOVA separately for the Visual Complex and the Auditory Complex tasks. As for the KSS questionnaire, a Time (1:9) x Group (iAG, cAG, ctrlAG) rANOVA was performed. A-priori hypotheses that RT, Accuracy, as well as the amplitudes of P3a and P3b ERP components are altered by HDTBR exposure and that the impact of HDTBR on cognitive task performance is affected by task complexity were checked with planned comparisons of least square means (contrasts). Therefore, instead of using the Time main effect, sessions were grouped into BDC (BDC-8, BDC-4), HDT (HDT3, HDT50 for the first campaign and HDT4, HDT49 for the second campaign), and R Phases (R + 2, R + 7, R + 13). Please note that the HDT Phase was limited to those HDT sessions taking place before centrifugation to assess the effect of the HDTBR without the potential effects of acute AG exposure. The same set of contrasts was applied to all outcome variables. The difference between pre-, and post-centrifugation sessions (Assessment time), and the moderation effect of the AG on HDTBR was also investigated with planned contrasts.

Huynh-Feldt correction was applied for all repeated measures with greater than 1 degree of freedom. Uncorrected degrees of freedom and corrected *p* values are reported. Partial eta squared (*ɳ*_p_^2^) was computed as an estimate of effect size.

Additionally, to estimate whether the learning curve for RT is affected differentially between HDTBR and AMBCtrl conditions, a linear model approach was chosen and was fitted on the data using all 9 sessions. The equation of the linear function was *y* = *mx* + *b*, where *y* represents RT, and *x* stands for session number. RT slopes (*m*), which represent the steepness and direction of the practice effect, were calculated separately for all four tasks for the HDTBR experimental groups (iAG, cAG, ctrlAG averaged together based on the lack of RT difference between the three groups) and for the AMBCtrl group. As a next step, RT slopes were compared between the HDTBR groups and the AMBCtrl group based on task modality (Visual, Auditory), and task complexity (Simple, Complex) to detect differences in the learning curve. A planned comparison of least square means with polynomial linear contrasts was used to analyze the slope difference between HDTBR and AMBCtrl conditions.

Further analyses regarding the three HDTBR experimental groups, including descriptive statistics, results for the aforementioned omnibus rANOVA analyses, planned comparisons for Phase (HDT, R), and Phase (HDT, R) x Complexity (Simple, Complex) interactions as well as results regarding the KSS questionnaire are presented in the Supplementary Notes, Supplementary Tables 1–11, and Supplementary Tables 21–23. All relevant analyses were also performed on the AMBCtrl group and are also presented in the Supplementary Notes and Supplementary Tables 12–20. Please note that only significant Supplementary results are mentioned in the main text.

### Reporting summary

Further information on research design is available in the Nature Research Reporting Summary linked to this article.

## Results

### The effect of HDTBR on cognitive functioning

To investigate the potential detrimental effects of 6° HDTBR on cognitive performance, pairwise comparisons were conducted for the three Phases (BDC, HDT, and R) in the HDTBR groups. This section details the comparisons between BDC and HDT and between BDC and R regarding RT, Accuracy, P3a, and P3b. Comparisons for HDT with R for all four variables are detailed in the Supplementary Notes, Supplementary Table 3, and Supplementary Table 7.

**Fig. 2:**
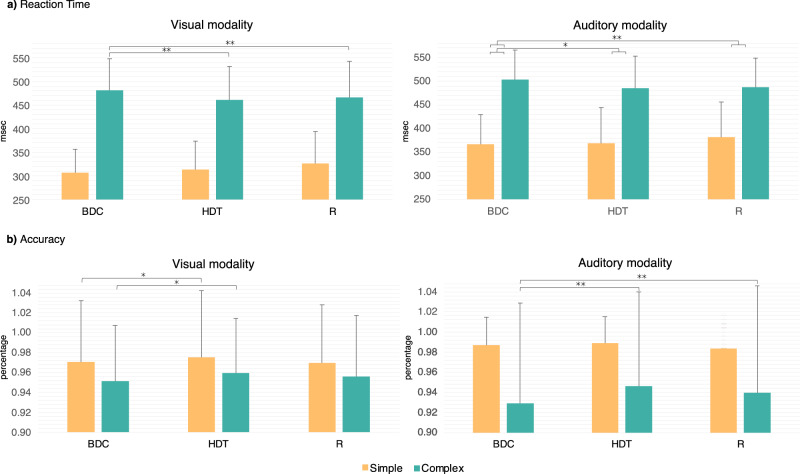
Behavioral task performance in the three HDTBR experimental groups (iAG, cAG and ctrlAG averaged together). **a** RT in the HDTBR groups (iAG, cAG and ctrlAG averaged together) comparing the three Phases (BDC, HDT and R). Regarding Simple tasks, a significant RT change was only present in the Auditory modality between the indicated Phases, while for the Complex tasks, a significant difference was found in both the Visual and the Auditory modality between the indicated Phases. **b** Accuracy in the HDTBR groups (iAG, cAG and ctrlAG averaged together) comparing the three Phases (BDC, HDT and R). The Simple and the Complex tasks are shown separately. A significant difference in Accuracy was only present in the Auditory modality and was limited to the Complex task between the indicated Phases. Error bars represent standard deviation (SD). *p < 0.05; **p < 0.01.

In general, RT was longer in the Complex tasks compared to Simple tasks in both modalities (for detailed descriptive statistics see Supplementary Table 1). Planned comparisons were used to test whether there was a significant change in RT between BDC and later phases in either the Visual or the Auditory modality (Table 1). Regarding the Visual modality, even though no significant overall Phase difference was obtained between BDC and HDT, the Phase (BDC, HDT) x Complexity (Simple, Complex) interaction was significant, as RT significantly decreased from BDC to HDT in the Visual Complex task, but not in the Visual Simple task. The BDC and R Phase comparison was also non-significant, however, again, the Phase (BDC, R) x Complexity (Simple, Complex) interaction was significant. Likewise, RT significantly decreased from BDC to R in the Visual Complex task, while it did not change in the Visual Simple task. Regarding the Auditory modality (Table 1) no significant overall Phase difference was found between BDC and HDT or between BDC and R. Once again, the Phase (BDC, HDT) x Complexity (Simple, Complex) and the Phase (BDC, R) x Complexity (Simple, Complex) interactions were both significant, as compared to BDC, RT decreased in the Auditory Complex task and increased in the Auditory Simple task during both HDT and R (Fig. 2a and Table 1).

**Table 1:** Results of the planned contrasts regarding RT and Accuracy for Phase effects (BDC vs HDT, and BDC vs R), and Phase × Complexity interactions in the HDTBR experimental groups (iAG, cAG and ctrlAG averaged together)

Measure	Modality	Effect	Df	*F*	*p*	Partial *η*2
RT	Visual	BDC, HDT	1(19)	1.468	0.240	0.072
		BDC, HDT × Simple, Complex	1(19)	37.042	<0.001**	0.061
		BDC, R	1(19)	0.083	0.776	0.004
		BDC, R × Simple, Complex	1(19)	14.803	0.001**	0.438
	Auditory	BDC, HDT	1(19)	1.809	0.194	0.087
		BDC, HDT × Simple, Complex	1(19)	4.459	0.048*	0.190
		BDC, R	1(19)	0.0005	0.981	0.000
		BDC, R × Simple, Complex	1(19)	16.791	<0.001**	0.469
ACC	Visual	BDC, HDT	1(19)	4.421	0.049*	0.189
		BDC, HDT × Simple, Complex	1(19)	0.620	0.440	0.032
		BDC, R	1(19)	0.197	0.662	0.010
		BDC, R × Simple, Complex	1(19)	2.513	0.129	0.117
	Auditory	BDC, HDT	1(19)	14.705	0.001**	0.436
		BDC, HDT × Simple, Complex	1(19)	13.982	0.001**	0.424
		BDC, R	1(19)	3.157	0.091	0.142
		BDC, R × Simple, Complex	1(19)	8.684	0.008**	0.314

*RT* reaction time, *ACC* accuracy, *BDC* baseline data collection phase, *HDT* head-down tilt phase, *R* recovery phase, *Df* degree of freedom, *F* regression mean square; *p < 0.05; **p < 0.01.

Results are presented based on task modality (Visual, Auditory), and task complexity (Simple, Complex).

In summary, modality-specific differences were revealed regarding the Simple tasks. While no significant change was present in the Visual Simple task, RT was increased in the Auditory Simple task and decreased in both the Visual and the Auditory Complex task during both HDT and R.

**Fig. 3:**
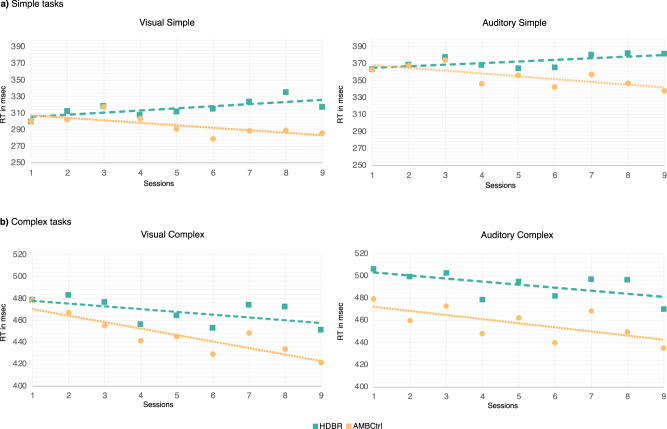
The practice effect in RT was estimated by a linear model fitted on all 9 sessions. The linear function equation was *y* = *mx* + *b*, (where *y* is the RT, *x* is the session number, and *m* is the slope of the RT), which was calculated separately for all four tasks for the HDTBR experimental groups (iAG, cAG, and ctrlAG averaged together) and for the AMBCtrl group. **a** Simple tasks, with increased RT in the HDTBR and decreased RT in the AMBCtrl in both Modalities. **b** A decreasing trend in the Complex tasks in both Modalities for the HDTBR and the AMBCtrl.

To estimate groupwise differences in the practice effect between the HDTBR groups and the AMBCtrl group, the practice effect on RT was characterized by the slope of a trend line estimated by linear regression for each task using session numbers (1–9) as a regressor. In the HDTBR groups, the slope was positive for the Simple tasks, while it was negative for the Complex tasks. As for the AMBCtrl group, RT showed a decreasing trend in all four tasks (Fig. 3 and Table 2).

**Table 2:** Slopes of the RT trend lines representing the practice effect in all four tasks for the HDTBR experimental groups (iAG, cAG and ctrlAGs averaged together) and for the AMBCtrl group

Modality	Complexity	HDTBR groups	AMBCtrl group
Visual	Simple	2.598	−3.026
	Complex	−2.513	−5.882
Auditory	Simple	1.902	−3.332
	Complex	−2.718	−3.732

HDTBR and AMBCtrl conditions were compared using planned comparison for least square means for each task separately (Table 3). A significant difference was found for the RT slopes regarding the Simple tasks in both Modalities as RT was increasing in the HDTBR groups, while it was decreasing in the AMBCtrl group. As for the Complex tasks, the slope was negative for both Modalities, indicating a modality-independent general RT decrease for the Complex tasks. A significant difference between the HDTBR groups and the AMBCtrl group was only found in the Visual Complex task suggesting a more pronounced practice effect in the AMBCtrl group, while in the Auditory Complex task, there was no difference between the AMBCtrl and the HDTBR conditions.

**Table 3:** Results for comparing the slopes of RT trend lines between the HDTBR experimental groups (iAG, cAG and ctrlAG averaged together) and the AMBCtrl group

Modality	Complexity	Comparison	D*f*	*F*	*p*	Partial *η*^2^
Visual	Simple	HDTBR, AMBCtrl	1(29)	6.283	0.018*	0.178
	Complex	HDTBR, AMBCtrl	1(29)	5.268	0.029*	0.154
Auditory	Simple	HDTBR, AMBCtrl	1(29)	4.985	0.033*	0.147
	Complex	HDTBR, AMBCtrl	1(29)	0.800	0.378	0.027

*HDTBR* head-down tilt bedrest, *AMBCtrl* ambulatory control, D*f* degree of freedom, *F* regression mean square; *p < 0.05; **p < 0.01.

Results are presented for both task modality and complexity.

Accuracy was generally higher in the Simple tasks compared to the Complex tasks in both modalities (for detailed descriptive statistics see Supplementary Table 4). Planned comparisons were used to test whether there was a significant change in Accuracy between BDC and later phases in either the Visual or the Auditory modality (Table 1 and Fig. 2b). In the Visual modality, there was a significant increase in Accuracy between BDC and HDT, but the Phase (BDC, HDT) × Complexity (Simple, Complex) interaction was not significant. The BDC and R Phase comparison and the Phase (BDC, R) × Complexity (Simple, Complex) interaction showed no significant differences. In the Auditory modality, a significant increase was revealed from BDC to HDT. A significant Phase (BDC, HDT) × Complexity (Simple, Complex) interaction was found with increased accuracy in the Auditory Complex task, and intact accuracy in the Auditory Simple task. The BDC to R Phase comparison showed no significant difference, but the Phase (BDC, R) × Complexity (Simple, Complex) interaction was significant with, again, increased accuracy in the Auditory Complex task and intact accuracy in the Auditory Simple task.

In summary, accuracy was significantly increased in both Visual Simple and Visual Complex tasks between BDC and HDT, but returned to baseline level for R. As for the Auditory modality, compared to BDC, increased accuracy was present in the Auditory Complex task during both HDT and R, while accuracy was intact in the Auditory Simple task during all three Phases.

**Fig. 4:**
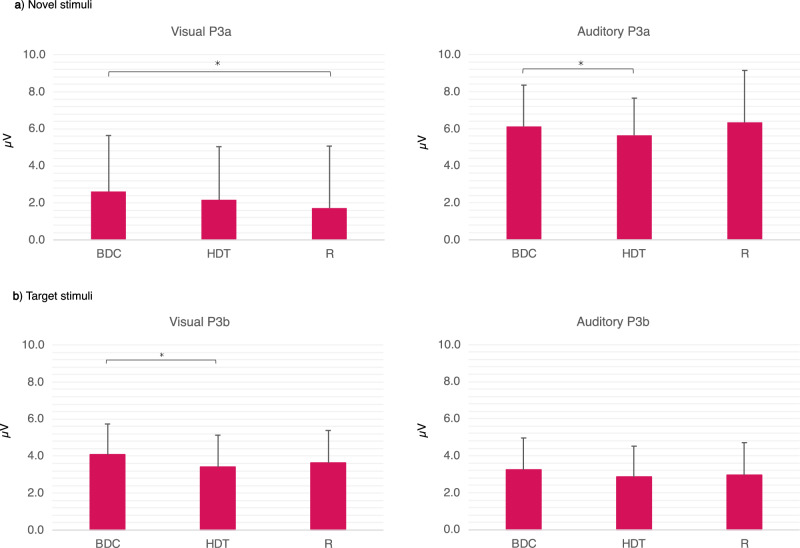
Amplitudes of ERP components (P3a and P3b) in the HDTBR experimental groups (iAG, cAG and ctrlAG averaged together). Complex tasks are analyzed in both modalities. **a** P3a amplitude (Irrelevant stimuli) in the HDTBR groups comparing the three Phases (BDC, HDT and R). **b** P3b amplitude (Target stimuli) in the HDTBR groups comparing the three Phases (BDC, HDT and R). Error bars represent standard deviation (SD). *p < 0.05; **p < 0.01.

As described earlier in the Data analysis section, P3a and P3b amplitudes were only analyzed for the Complex tasks (Table 4). Regarding the Visual P3a amplitude, planned comparisons showed no significant change between BDC and HDT, however, there was a significant decrease from BDC to R. While for the Auditory P3a amplitude, there was a significant decrease between BDC and HDT, but no difference was shown between the BDC and R (Fig. 4a).

**Table 4:** Results of the planned contrasts regarding the ERP amplitude of P3a and P3b during Complex tasks for Phase effects (BDC vs HDT, and BDC vs R) in the three HDTBR experimental groups (iAG, cAG, and ctrlAG averaged together)

Measure	Modality	Effect	D*f*	*F*	*p*	Partial *η*^2^
P3a	Visual	BDC, HDT	1(19)	2.086	0.164	0.099
		BDC, R	1(19)	4.525	0.047*	0.192
	Auditory	BDC, HDT	1(19)	4.478	0.047*	0.191
		BDC, R	1(19)	0.314	0.581	0.016
P3b	Visual	BDC, HDT	1(19)	5.712	0.027*	0.231
		BDC, R	1(19)	4.186	0.055	0.181
	Auditory	BDC, HDT	1(19)	4.274	0.053	0.184
		BDC, R	1(19)	2.012	0.172	0.096

*BDC* baseline data collection phase, *HDT* head-down tilt phase, *R* recovery phase, D*f* degree of freedom, *F* regression mean square; *p < 0.05; **p < 0.01.

Results are presented based on task modality (Visual, Auditory).

As for the Visual P3b amplitude, a significant decrease was only revealed between BDC and HDT, but not between BDC and R. On the contrary, for the Auditory P3b amplitude, no significant change was detected between BDC and HDT, or between BDC and R (Fig. 4b).

In summary, there were modality-specific differences regarding both P3a and P3b amplitudes.

### The acute effects of the AG protocol

This section aimed to investigate the acute effects of different AG protocols in two HDTBR groups exposed to intermittent (iAG) and continuous (cAG) AG. Analyses for the acute effect of AG are based on the Assessment time comparisons between the pre-and post-centrifugation measurements performed on consecutive days.

Using planned comparison, no significant Acute AG effect (pre-, post-centrifugation) was revealed regarding RT in the Visual or the Auditory tasks (Table 5). Neither the interaction between the Acute AG effect and AG protocols (Assessment time (pre-centrifugation, post-centrifugation) × AG Type (iAG, cAG)), nor the interaction between Acute AG effect and Complexity (Assessment time (pre-centrifugation, post-centrifugation) × Complexity (Simple, Complex)) were significant. In summary, neither AG as such nor AG Type had any significant Acute effect on RT in the Visual or Auditory modality.

**Table 5:** Results of the planned contrasts regarding the behavioral data of the HDTBR experimental groups (iAG, cAG and ctrlAG averaged together)

Measure	Modality	Effect	D*f*	*F*	*p*	Partial *η*^2^
RT	Visual	Pre-, Post	1(19)	0.349	0.561	0.018
		Pre-, Post × AG Type (iAG, cAG)	1(19)	0.975	0.335	0.049
		Pre-, Post × Simple, Complex	1(19)	0.051	0.822	0.003
	Auditory	Pre-, Post	1(19)	1.117	0.303	0.056
		Pre-, Post × AG Type (iAG, cAG)	1(19)	0.079	0.780	0.004
		Pre-, Post × Simple, Complex	1(19)	2.946	0.102	0.134
ACC	Visual	Pre-, Post	1(19)	2.979	0.100	0.136
		Pre-, Post × AG Type (iAG, cAG)	1(19)	1.782	0.197	0.086
		Pre-, Post × Simple, Complex	1(19)	0.709	0.410	0.036
	Auditory	Pre-, Post	1(19)	1.711	0.206	0.083
		Pre-, Post × AG Type (iAG, cAG)	1(19)	1.144	0.298	0.057
		Pre-, Post × Simple, Complex	1(19)	2.171	0.156	0.103
P3a	Visual	Pre-, Post	1(19)	0.048	0.828	0.003
		Pre-, Post × iAG, cAG	1(19)	0.998	0.330	0.050
	Auditory	Pre-, Post	1(19)	1.350	0.259	0.066
		Pre-, Post × iAG, cAG	1(19)	1.934	0.180	0.092
P3b	Visual	Pre-, Post	1(19)	3.051	0.097	0.138
		Pre-, Post × iAG, cAG	1(19)	0.002	0.966	0.000
	Auditory	Pre-, Post	1(19)	0.083	0.776	0.004
		Pre-, Post × iAG, cAG	1(19)	0.208	0.654	0.011

*RT reaction time, ACC accuracy, Pre* pre-centrifugation measurement, *Post* post-centrifugation measurement, *iAG* intermittent artificial gravity group, *cAG* continuous artificial gravity group, *Df* degree of freedom, *F* regression mean square.

Reaction time (RT) and accuracy (ACC) for assessment time (pre- and post-centrifugation), assessment time × complexity, and assessment time × AG type (iAG, cAG) interactions. Results are presented based on task modality (Visual, Auditory).

Regarding Accuracy in the Visual Simple and Complex tasks, the planned comparison showed no significant effect of Assessment time (pre-, post-centrifugation) (Table 5). In line with that, the Assessment time (pre-, post-centrifugation) × AG Type (iAG, cAG), and the Assessment time (pre-, post-centrifugation) × Complexity (Simple, Complex) interactions were both non-significant. A similar pattern was detected in the case of the Auditory Simple and Complex tasks (Table 5). In summary, AG and AG protocol type had no Acute effect on Accuracy in the Visual or Auditory modality.

In terms of Visual as well as Auditory P3a amplitude, no Acute effect of AG was found (Table 5). The planned comparison showed no significant effect of Assessment time (pre-, post-centrifugation). There was no evidence in favor of any of the AG types (Assessment time (pre-, post-centrifugation) × AG Type (iAG, cAG)). No Acute effect of AG was found regarding the Visual or Auditory P3b amplitude either. The planned comparison showed no significant effect of Assessment time (pre-, post-centrifugation), or the Assessment time (pre-, post-centrifugation) × AG Type (iAG, cAG) interaction.

### Examining the chronic effect of AG itself and AG protocol types

To determine whether AG exposure mitigated the potential negative effect of HDTBR, Phase effects of AG and AG protocol types were compared between BDC and HDT, and between BDC and R. Analyses for the Phase effects of AG include AG Groups (iAG, cAG) versus ctrlAG group comparisons, while analyses for Phase effects of AG protocol types only includes iAG versus cAG group comparisons.

There was no significant Phase effect of AG on RT in the Visual Simple and Complex tasks, as neither the Phase (BDC, HDT) × AG Exposure (AG Groups, ctrlAG) × Complexity (Simple, Complex), nor the Phase (BDC, R) × AG Exposure (AG Groups, ctrlAG) × Complexity (Simple, Complex) interactions were significant (Table 6). Also, no Phase difference was present between the two AG protocols, as the Phase (BDC, HDT) × AG Type (iAG, cAG) × Complexity (Simple, Complex), and Phase (BDC, R) × AG Type (iAG, cAG) × Complexity (Simple, Complex) interactions were also non-significant. Similarly, in the Auditory modality (Table 6), no Phase effect was found for RT regarding the AG exposure itself, or the AG protocol type, as none of the analyses yielded significant results.

**Table 6:** Results of the planned contrasts regarding the HDTBR groups regarding RT and ACC for Phase (BDC vs HDT, BDC vs R) × AG Exposure (AG Groups vs ctrlAG) × Complexity (Simple and Complex), and for Phase (BDC vs HDT, BDC vs R) × AG type (iAG, cAG) interactions

Measure	Modality	Effect	D*f*	*F*	*p*	Partial *η*^2^
RT	Visual	BDC, HDT × AG Groups, ctrlAG × Simple, Complex	1(19)	0.179	0.676	0.009
		BDC, R × AG Groups, ctrlAG × Simple, Complex	1(19)	0.248	0.623	0.013
		BDC, HDT × iAG, cAG × Simple, Complex	1(19)	0.017	0.898	0.001
		BDC, R × iAG, cAG × Simple, Complex	1(19)	0.791	0.385	0.040
	Auditory	BDC, HDT × AG Groups, ctrlAG × Simple, Complex	1(19)	0.001	0.968	0.000
		BDC, R × AG Groups, ctrlAG × Simple, Complex	1(19)	0.324	0.575	0.017
		BDC, HDT × iAG, cAG × Simple, Complex	1(19)	1.919	0.182	0.092
		BDC, R × iAG, cAG × Simple, Complex	1(19)	1.184	0.290	0.059
ACC	Visual	BDC, HDT × AG Groups, ctrlAG × Simple, Complex	1(19)	0.162	0.691	0.008
		BDC, R × AG Groups, ctrlAG × Simple, Complex	1(19)	11.567	0.002**	0.378
		BDC, HDT × iAG, cAG × Simple, Complex	1(19)	0.112	0.742	0.006
		BDC, R × iAG, cAG × Simple, Complex	1(19)	2.128	0.161	0.101
	Auditory	BDC, HDT × AG Groups, ctrlAG × Simple, Complex	1(19)	1.175	0.291	0.058
		BDC, R × AG Groups, ctrlAG × Simple, Complex	1(19)	0.104	0.750	0.005
		BDC, HDT × iAG, cAG × Simple, Complex	1(19)	0.557	0.465	0.028
		BDC, R × iAG, cAG × Simple, Complex	1(19)	0.947	0.343	0.047

*RT* reaction time, *ACC* accuracy, *AG Groups* artificial gravity groups (intermittent and continuous groups), *ctrlAG* control artificial gravity group, *iAG* intermittent artificial gravity group, *cAG* continuous artificial gravity group, *BDC* baseline data collection phase, *HDT* head-down tilt phase, *R* recovery phase, *iAG* intermittent artificial gravity group, *cAG* continuous artificial gravity group, *Df* degree of freedom, *F* regression mean square; *p < 0.05; **p < 0.01.

Results are presented based on task modality (Visual, Auditory).

In summary, no Phase effect of AG exposure was found between BDC and HDT, and between BDC and R for P3a and P3b regardless of task modality. Similarly, no differences were found between the two AG protocol types regarding P3a and P3b amplitudes regardless of task modality.

Examining AG as a countermeasure against HDTBR in the case of Visual modality (Table 6), the Phase (BDC, HDT) × AG Exposure (AG Groups, ctrlAG) × Complexity (Simple, Complex) interaction was non-significant. On the contrary, the Phase (BDC, R) × AG Exposure (AG Groups, ctrlAG) × Complexity (Simple, Complex) interaction was significant with a significantly increased Accuracy in the Visual Complex task and significantly decreased Accuracy in the Visual Simple task regarding the AG Groups, while no significant changes were seen in the ctrlAG group. The Complexity main effect was not significant (see in the Supplementary Notes and Supplementary Table 2). As for the AG protocol type comparisons, the Phase (BDC, HDT) × AG Type (iAG, cAG) × Complexity (Simple, Complex), and the Phase (BDC, R) × AG Type (iAG, cAG) × Complexity (Simple, Complex) interactions were both not significant. Regarding the Auditory modality, AG exposure itself and AG protocol type had no Phase effect on Accuracy, as none of the interactions showed significant differences.

In summary, Accuracy remained intact in the ctrlAG group as well as in the AG groups regardless of task modality or complexity between BDC and HDT. However, regarding the BDC and R Phase comparison, while Accuracy did remain intact in the ctrlAG group, there was a significant increment in the Visual Complex task and a significant decrement in the Visual Simple task in the AG groups. No significant Phase effects were present between BDC and HDT or BDC and R between the two AG protocol types regardless of task modality or complexity.

Investigating AG as a countermeasure against HDTBR regarding the Visual and Auditory P3a amplitude, Phase (BDC, HDT) × AG Exposure (AG Groups, ctrlAG) and the Phase (BDC, R) × AG Exposure (AG Groups, ctrlAG) interactions were non-significant for both modalities (Table 7). Comparing the effects of AG protocol types yielded similar results, as the Phase (BDC, HDT) × AG Type (iAG, cAG), and the Phase (BDC, R) × AG Type (iAG, cAG) interactions were also not significant. Regarding the Auditory P3b amplitude (Table 7), the Phase (BDC, HDT) × AG Exposure (AG Groups, ctrlAG), and the Phase (BDC, R) × AG Exposure (AG Groups, ctrlAG) interactions were also non-significant (Table 7). Comparing the effects of AG protocol types yielded similar results, as the Phase (BDC, HDT) × AG Type (iAG, cAG), and the Phase (BDC, R) × AG Type (iAG, cAG) interactions were also not significant.

**Table 7:** Results of the planned contrasts regarding the HDTBR groups regarding P3a, and P3b for Phase (BDC vs HDT, BDC vs R) × AG Exposure (AG Groups vs ctrlAG) × Complexity (Simple and Complex), and for Phase (BDC vs HDT, BDC vs R) × AG type (iAG, cAG) interactions

Measure	Modality	Effect	D*f*	*F*	p	Partial *η*^2^
P3a	Visual	BDC, HDT × AG Groups, ctrlAG	1(19)	0.002	0.957	0.000
		BDC, R × AG Groups, ctrlAG	1(19)	0.552	0.466	0.028
		BDC, HDT × iAG, cAG	1(19)	1.104	0.307	0.055
		BDC, R × iAG, cAG	1(19)	0.001	0.970	0.000
	Auditory	BDC, HDT × AG Groups, ctrlAG	1(19)	0.092	0.764	0.005
		BDC, R × AG Groups, ctrlAG	1(19)	0.196	0.662	0.010
		BDC, HDT × iAG, cAG	1(19)	0.341	0.566	0.018
		BDC, R × iAG, cAG	1(19)	0.466	0.503	0.024
P3b	Visual	BDC, HDT × AG Groups, ctrlAG	1(19)	0.360	0.555	0.019
		BDC, R × AG Groups, ctrlAG	1(19)	0.214	0.649	0.011
		BDC, HDT × iAG, cAG	1(19)	0.392	0.539	0.020
		BDC, R × iAG, cAG	1(19)	0.366	0.552	0.019
	Auditory	BDC, HDT × AG Groups, ctrlAG	1(19)	1.626	0.218	0.079
		BDC, R × AG Groups, ctrlAG	1(19)	0.115	0.739	0.006
		BDC, HDT × iAG, cAG	1(19)	0.000	0.986	0.000
		BDC, R × iAG, cAG	1(19)	0.245	0.627	0.013

*AG Groups* artificial gravity groups (intermittent and continuous groups), *ctrlAG* control artificial gravity group, *iAG* intermittent artificial gravity group, *cAG* continuous artificial gravity group, *BDC* baseline data collection phase, *HDT* head-down tilt phase, *R* recovery phase, *iAG* intermittent artificial gravity group, *cAG* continuous artificial gravity group, *Df* degree of freedom, *F* regression mean square; *p < 0.05; **p < 0.01.

Results are presented based on task modality (Visual, Auditory).

In summary, no Phase effect of AG exposure was found between BDC and HDT, and between BDC and R for P3a and P3b regardless of task modality. Similarly, no differences were found between the two AG protocol types regarding P3a and P3b amplitudes regardless of task modality.

## Discussion

The present study was designed to evaluate the Acute and Chronic effects of intermittent and continuous AG on cognitive performance and electrocortical activity during prolonged exposure to HDTBR, a ground-based space analog condition for microgravity-induced physiological changes^43,44^. To identify whether potential cognitive changes are specific to task modality and/or level of task complexity, tasks were presented in both the Auditory and the Visual modality with two levels of task complexity in each modality. While previous studies investigated visual information processing^1^, research is scarce regarding acoustic information processing during actual and simulated space conditions. Given that the ability to process visual and acoustic information properly is of equal importance during spaceflight, the inclusion of the Auditory modality in this study is of particular importance.

The first aim was to identify changes in cognitive performance related to HDTBR itself. Behavioral measures of task performance (RT, Accuracy) and electrophysiological measures of controlled and automatic attention (P3a and P3b) were investigated. Even though HDTBR was expected to worsen cognitive functioning^1^, no negative effect of HDTBR was found on behavioral performance during Complex tasks regardless of task modality. A negative effect of HDTBR was only present for the Auditory Simple task, which was limited to RT. Moreover, results even showed improved performance during and after HDTBR exposure in the Complex tasks. Our results are consistent with the literature investigating spatial working memory, attention, and psychomotor speed under HDTBR, as the majority reported unchanged^35,45^ or even improved^46^ performance under HDTBR (for a detailed description see^1^). The only exception is a study by Brauns et al. ^47^ which also showed intact RT and Accuracy but found worse efficiency (as calculated by the average of z-transformed accuracy and RT values) during and after 60-day HDTBR using a visual 3-stimulus oddball task. Even though the task used by Brauns et al. ^47^ shared some similarities with the Visual Complex task used in this study, their task was less demanding as no spatial working memory was involved. Additionally, there were only 3 measurement points, and the single HDTBR measurement point was scheduled at the very end of the 60-day-long HDTBR. Regarding the Auditory modality, a negative impact of HDTBR on behavioral measures was shown to be limited to RT in the Auditory Simple task, while improved Accuracy was found in the Auditory Complex task. However, our finding of decreased RT in the Auditory Simple task is hard to interpret on its own. To our knowledge, there is only one other study that has assessed cognitive processes in the Auditory modality during HDTBR. As a part of AGBRESA, Tays et al.^48^ investigated the effect of centrifugation on auditory information processing speed and flexibility using the Paced Auditory Serial Addition Test (PASAT). Compared to HDTBR control, they found increased accuracy and RT in the iAG and cAG groups during HDTBR, which is in line with current results regarding increased Accuracy in the Auditory Complex task. However, there were no pre-post-HDTBR comparisons and no simple Auditory task, making our finding of decreased RT in the Auditory Simple task hard to compare to related literature. This underscores the necessity for further investigations in this domain.

In addition to the above-described behavioral measures, the current study also explored the potential impact of HDTBR on the practice effects related to repeated task administration by comparing the learning curves for RT throughout the nine sessions between the HDTBR experimental groups with the AMBCtrl group. A difference was found regarding the direction and the steepness of the RT slopes for the Visual Simple task with increased RT in the HDTBR groups and decreased RT in the AMBCtrl group. As for the Visual Complex task, while the HDTBR and the AMBCtrl groups both showed RT decrement, it was more pronounced in the AMBCtrl group. Similar to the Visual Simple task, a difference was also found for the direction and the steepness for the RT slopes of the Auditory Simple task with increased RT in the HDTBR groups and decreased RT in the AMBCtrl group, while there was no groupwise difference regarding the Auditory Complex task. Taken together, despite having intact RT and Accuracy in the Visual Simple task and improved RT and Accuracy in the Visual Complex task, the reduced practice effect suggests disrupted learning processes during prolonged HDTBR exposure in the Visual modality. Regarding the Auditory modality, while the Auditory Complex task seems to be unaffected by HDTBR on the behavioral level, a clear performance deterioration was detected in the Auditory Simple task with increased RT and reduced practice effect during and after HDTBR. Disrupted learning processes without behavioral deterioration during HDTBR have already been shown in the Visual modality. Lipnicki et al.^49^ reported improved performance during and after 60 days of HDTBR exposure in a visual 2-back and the Flanker task along with a decreased practice effect compared to the ambulatory control group. As a greater improvement was found in working memory task performance in the ambulatory control group compared to the bedrest group, they also concluded that bedrest may attenuate the learning effect. Our study is the first to demonstrate clear, modality-independent deterioration of the practice effect during HDTBR in both the Visual and Auditory modalities regarding Simple tasks. This may be due to the fact that repetitive and simple tasks are known to induce lapses of attention and time-on-task effects^50^.

Along with behavioral measures, this study also investigated the effect of HDTBR exposure on late positive ERP components P3a and P3b. Compared to BDC, the P3a amplitude remained intact during HDTBR in the Visual modality while being decreased in the Auditory modality. The opposite was observed for the P3b amplitude, as it was decreased during HDTBR in the Visual modality while being intact in the Auditory modality. During HDTBR, P3a amplitude was only decreased in the Auditory Complex task, which might imply modality-specific disturbance of automatic attentional processes, while the P3b decrement possibly suggests impaired controlled attentional processes in the Visual modality. However, both Auditory P3a and Visual P3b amplitudes did return to the baseline level for R suggesting that the observed HDTBR-related changes are not persistent. Very few studies have investigated the effect of HDTBR on event-related potentials, and no studies are yet available concerning the Auditory P3a and P3b. Out of the two available studies, one investigated the impact of prolonged HDTBR exposure on emotional picture processing^51^. They performed a groupwise comparison between participants undergoing 60 days of HDTBR with matched controls at day 30 of HDTBR exposure. While there was no difference regarding neutral picture processing and behavioral measures between the two groups, HDTBR group members showed smaller Visual P3b and smaller late positive potential (LPP) amplitudes for pleasant and unpleasant emotional stimuli. The other study explored the impact of HDTBR and antioxidant supplementation on attentional processing^47^. They found reduced Visual P3a and P3b amplitudes during the last phase of 60-day-long HDTBR exposure and post-HTDBR using a 3-stimulus oddball task. As mentioned earlier, the study of Brauns et al.^47^ only included 3 measurement points and the single HDTBR measure was scheduled on the very last day of HDTBR exposure. While P3a and P3b amplitudes remained reduced even after HTBRT in the study of Brauns et al.^47^, the reduced Visual P3b amplitude did return to baseline level in the current study. Such a discrepancy between results may either be attributed to differences between the studies regarding the timing of the assessments which may contribute to different levels of task habituation between the two studies or the difference regarding task complexity. As for the possible modality specific impact of HDTBR on P3a and P3b found in our study, additional research is required in the Auditory domain to elucidate the underlying reason for these discrepancies.

Even though no clear complexity or modality-specific HDTBR-related changes were shown regarding behavioral or ERP parameters, results do indicate that HDTBR may have a negative impact on cognitive performance. Current findings might be at least partially attributed to alteration of the neuroelectric processing due to HDTBR-induced cephalic fluid shift and upward shift of the brain^15,52^, which has already been shown during prolonged HDTBR^47^. An important aim of the present study was to utilize the HDTBR-induced changes in spatial working memory and attention as an indicator of how similar physiological alterations (e.g. cephalad fluid shift and the upward brain shift) during spaceflight could impair these cognitive functions. Three space studies have already targeted visual working memory using a line orientation task (a case study of a 6-day-long space mission^53,54^, and 2 half-year-long space missions^10,55^. While this is the first such study in HDTBR, the significance of the present research is the application of the same Visual Complex task that has been used in the previous space study by Takács et al.^9^, making results directly comparable regarding the Visual modality. Takács et al. ^9^ found behavioral performance deterioration along with Visual P3a and P3b amplitude decrements during the earlier and later stages of the mission and a few days after returning to Earth. Even though task performance did return to preflight level, ERP amplitudes remained diminished even 2–3 weeks into the post-flight period, reflecting slow re-adaptation to terrestrial conditions. Contrary, current results show intact Visual P3a and decreased Visual P3b amplitudes during 60 days of HDTBR along with improved behavioral performance during the Visual Complex task, while no pre-post HDTBR difference was shown for either behavioral or ERP measures. However, compared to ambulatory controls, reduced practice effects do indicate disrupted learning processes during prolonged HDTBR exposure. The divergence of the results between HDTBR and spaceflight and the fact that spaceflight has a more clear negative impact on cognitive performance might be attributed to those space-related stressors that are not present during bedrest (e.g. cosmic radiation, elevated CO_2_ levels, altered light exposure) and additional stressors that are less or not present during bedrest (e.g. high workload, isolation, and confinement)^2,56,57^. Another possibility is that besides cephalad fluid shift other factors contributed to decreased Visual P3b and Auditory P3a amplitudes during HDTBR. One such factor could be immobilization during bedrest^58^. Showing similar P3a and P3b amplitude changes during HDTBR, Brauns et al.^47^ argued that physical inactivity during bedrest can result in reduced dopamine and norepinephrine concentrations, which are suggested to play a role in P3a^25,59^ and P3b generation^60,61^ respectively. Therefore, bedrest-related alterations in the neurotransmitter system might also play a role in the observed changes.

In search of an appropriate countermeasure protocol for future space missions, the present study also aimed to evaluate the Acute and Chronic effect of intermittent and continuous short-arm centrifugation on cognitive processes during 60 days of HDTBR. Results revealed no significant Acute impact of AG protocols on behavioral performance or ERP parameters regardless of task complexity and modality and there was no difference between the continuous and intermittent AG groups in this matter. The findings of the present study are in line with the results of previous studies focusing on the acute effect of AG. Schneider et al.^62^, for example, recorded EEG during and after a single bout of 15 min long AG exposure. Even though increased neural activity was found in the prefrontal cortex during AG, it reached baseline levels immediately after centrifugation. Dern et al.^63^ recorded EEG before, during, and after 30 min of intermittent and continuous AG, cognitive performance (including executive functions, decision-making, information processing, and working memory) and self-reported mood assessment were also administered before and after AG exposure. Their results show no evidence that centrifugation would acutely disturb cognitive performance and found no difference between 30 min of intermittent and continuous AG exposure protocols in this matter, supporting the findings of the current study.

Regarding the Chronic effects of AG, the potential negative impact of HDTBR on cognitive functioning was predicted to be less pronounced in the two experimental groups receiving AG. When comparing the two AG groups with the ctrlAG, results showed no long-term impact of AG protocols on RT between BDC and HDT or BDC and R regardless of task complexity and modality. Accuracy also remained intact in the AG groups regardless of task complexity or modality between BDC and HDT. However, there was an increment in the Visual Complex task and a decrement in the Visual Simple task in the AG groups between BDC and R, while no such changes were observed in the ctrlAG group. P3a and P3b amplitudes were also not affected by AG exposure either in the Visual or the Auditory modality. Additionally, it was also evaluated whether intermittent and continuous centrifugation protocols affect the cognitive system differently, but no difference was revealed between AG protocols. AG exposure also had no impact on subjective sleepiness as measured by KSS as there was no group difference between the three experimental groups. As a conclusion, the results of this study show that intermittent or continuous AG types have a negligible effect on the Visual modality and no effect on the Auditory modality regarding cognitive task performance. As part of the AGBRESA project, Basner et al.^32^ and Tays et al.^48^ also investigated cognitive performance of the same set of participants taking part in this study. Basner et al.^32^ administered NASA’s Cognition battery along with alertness and mood surveys repeatedly before, during, and after HDTBR. Even though they did find a modest slowing in cognitive behavioral performance and decrement in emotional recognition in all three HDTBR groups, these changes were not mitigated by AG exposure as no difference in performance was detected between the intermittent and continuous AG groups. The only significant difference was that participants in the intermittent and continuous AG groups reported higher workload compared to the HDTBR control group (ctrlAG). Tays et al.^48^ also found no HDTBR-related differences in cognition, balance, and functional mobility between the three HDTBR groups. Post-hoc comparisons revealed that participants of the intermittent AG group reported less severe motion sickness symptoms compared to the continuous AG group during centrifugation, and the level of motion sickness in the intermittent AG group was similar to those not receiving AG. A further advantage of the intermittent AG was that the duration of post-centrifugation illusory motion was shorter in this group compared to the continuous AG. Another study taking part in the AGBRESA project by Frett et al.^22^ compared the tolerability of the two centrifugation protocols on cardiovascular load and motion sickness. Mean heart rates were revealed to be affected by the time spent in HDTBR for the continuous AG group, but not for the intermittent AG group. In line with the results of Tays et al. ^48^, Frett et al.^22^ found the daily assessed motion sickness scores to be significantly higher in the continuous AG group regardless of the number of days spent in HDTBR, while no differences were observed between groups in subjective exertion, mood or sleepiness. Authors concluded that both protocols are tolerable regarding cardiovascular loading and motion sickness, with the intermittent centrifugation causing marginally fewer symptoms. While this study found no differences between the two AG protocols, it also confirms no detrimental effects or improvements related to AG.

As a proper countermeasure protocol for future deep space missions should not only be able to serve against microgravity-induced physiological alterations, but it should also present no harm to astronauts’ cognitive capacity, results of this study support the use of both AG protocols as a potential countermeasure for prolonged space missions. The strength of this study involves a highly controlled and systematic study design with multiple data collection points, and various cognitive tasks enabling investigation of the impact of HDTR and AG on task modality and level of task complexity. The study design also allowed direct comparability with space-related results and being part of the AGBRESA project also enabled direct comparison of the countermeasure efficacy among bedrest studies following the same study protocol^31^. However, this study also has some limitations. The sample size and the unbalanced gender composition of the experimental groups limit the generalizability of the findings. As discussed in another recent AGBRESA study^22^, the sample size does not enable well-powered statistical analyses about gender differences. Gender should be taken into consideration for future studies, to fully understand the neurocognitive effects of HDTBR and AG. Moreover, increasing the sample size per group is recommended to enhance the generalizability of the behavioral findings.

In conclusion, our study confirmed that HDTBR-induced factors may negatively affect attentional functions and working memory in the Visual and Auditory modalities. The present study is a significant contribution to the HDTBR literature demonstrating that besides visual information processing, acoustic information processing can also be impaired during bedrest. Furthermore, our results also show that neither of the two AG protocol types had any Acute or Chronic effect on the investigated cognitive measures, making them equally promising candidates as countermeasures against some of the main detrimental effects of microgravity. It is also important to underline that while AG is suitable for hindering bone loss, cardiovascular deconditioning, muscle weakening, regulatory disorders, and sensorimotor and neuro-vestibular disturbances, it is not able to act against radiation exposure, altered circadian rhythm, and psychological challenges associated with prolonged confinement and isolation^18^. Therefore, the search for complementary countermeasures enabling the full safety of long-duration space travel is still on.

### Supplementary information

Supplementary Information

Reporting Summary

## Data Availability

Data underlying statistical analyses are available at https://figshare.com/projects/NeuroGravity/161770.

## Code availability

The analyzed EEG and behavioral data tables and Matlab scripts supporting the findings of this study are available at figshare with the identifier(s) [data table: 10.6084/m9.figshare.22246096.v1, scripts: 10.6084/m9.figshare.22246138.v1].
