# Supplementary material for: Effect of artificial gravity on neurocognitive performance during head-down tilt bedrest
Source: NPJ Microgravity. 2024 Jun 5;10:59. doi: 10.1038/s41526-024-00405-4 (PMC11153507; doi:10.1038/s41526-024-00405-4)
Supplement: Supplementary file 1 — Supplementary Information [file 41526_2024_405_MOESM1_ESM.pdf]

Effect of artificial gravity on neurocognitive performance during head-down tilt bedrest

Running head: Cognitive performance during HDTBR and Artificial Gravity

Authors: Borbála Tölgyesi[1,2,*,†], Anna Altbäcker[1†], Irén Barkaszi[1], Tim Stuckenschneider[3,4], Leonard Braunsmann[3], Endre Takács[1], Bea Ehmann[1], László Balázs[1], Vera Abeln[3]

Affiliations:

[1] Institute of Cognitive Neuroscience and Psychology, HUN-REN Research Centre for Natural Sciences, Budapest, Hungary

[2] Interaction and Immersion Hub, Innovation Center, Moholy-Nagy University of Art and Design, Budapest, Hungary

[3] Institute of Movement and Neurosciences, Centre for Health and Integrative Physiology in Space (CHIPS), German Sport University Cologne, Germany

[4] Geriatric Medicine, Department for Health, Services Research, School of Medicine and Health Sciences, Carl von Ossietzky University, Oldenburg, Germany

[†] These authors have contributed equally to this work and share first authorship.

* Corresponding author. Email: annaaltbacker@gmail.com

Supplementary Information

Supplementary Notes

The supplementary includes additional analyses for RT, Accuracy, ERP components P3a, and P3b, and results for the Karolinska Sleepiness Scale (KSS) questionnaire. Here we report descriptive statistics for all variables, results for all performed rANOVAs, and planned comparisons for an additional Phase effect (HDT, R), and Phase effect (HDT, R) x Complexity (Simple, Complex) interactions regarding the three HDTBR groups. All relevant analyses for the HDTBR groups were also performed for the AMBCtrl group and are also detailed here.

Supplementary results regarding the effect of HDTBR on cognitive functioning are presented first, including descriptive statistics, results for rANOVAs, planned comparisons for HDT versus R Phase effect, and Phase (HDT, R) x Complexity (Simple, Complex) interaction are reported for RT, Accuracy, and ERP components (P3a and P3b) for the three HDTBR experimental groups. In addition, results for the KSS questionnaire in the three experimental HDTBR groups are also detailed here.

Descriptive statistics for RT are presented in Supplementary Table 1. As for the rANOVA results for RT in the Visual tasks, the rANOVA (Time (1:9) x Complexity (Simple, Complex) x Group (iAG, cAG, ctrlAG)) revealed non-significant Time and Group main effects (see Supplementary Table 2). While the Complexity main effect was significant with RT being longer in the Visual Complex Task compared to the Visual Simple Task, no significant Time x Group interaction was obtained. As for the (HDT, R) Phase effect, planned comparisons showed no significant difference in RT, and the Phase (HDT, R) x Complexity (Simple, Complex) interaction was also not significant (Supplementary Table 3).

A similar pattern was present for RT in the Auditory tasks. The rANOVA (Time (1:9) x Complexity (Simple, Complex) x Group (iAG, cAG, ctrlAG)) showed non-significant Time and Group main effects (Supplementary Table 2). Just as in the Visual Tasks, the Complexity main effect was significant with RT being longer in the Auditory Complex Task compared to the Auditory Simple Task while no significant Time x Group interaction was obtained. Again, there was no significant Phase difference between HDT and R, and the Phase (HDT, R) x Complexity (Simple, Complex) interaction was also not significant (Supplementary Table 3).

Descriptive statistics for Accuracy are presented in Supplementary Table 4. Regarding Accuracy in the Visual Tasks, the rANOVA (Time (1:9) x Complexity (Simple, Complex) x Group (iAG, cAG, ctrlAG)) showed a non-significant Time main effect, and non-significant Group main effect (see Supplementary Table 2). The Complexity main effect was significant, with Accuracy being significantly higher in the Visual Simple Task compared to the Visual Complex Task, while the Time x Group interaction was non-significant. Planned comparisons showed no significant Phase effect between HDT and R, and the Phase (HDT, R) x Complexity (Simple, Complex) interaction was also not significant (Supplementary Table 3).

Just as in the Visual tasks, the rANOVA (Time (1:9) x Complexity (Simple, Complex) x Group (iAG, cAG, ctrlAG)) showed no significant Time or Group main effect for the Auditory Tasks (see Supplementary Table 2). Once again, the Complexity main effect was significant, with Accuracy being significantly higher in the Auditory Simple Task compared to the Auditory Complex Tasks. The Time x Group interaction was not significant. As for the planned comparisons, no significant difference was found between HDT and R, and the Phase (HDT, R) x Complexity (Simple, Complex) interaction was also not significant (Supplementary Table 3).

Descriptive statistics for P3a and P3b amplitudes are presented in Supplementary Table 5. According to the rANOVA (Time (1:9) x Group (iAG, cAG, ctrlAG)), the Time main effect was significant, with Visual P3a amplitude decrement over Time, but there was no significant Group main effect (Supplementary Table 6). The Time x Group interaction was also not significant. Additionally, the planned comparison showed no significant Phase effect between HDT and R (Supplementary Table 7).

Regarding the Auditory P3a amplitude, the rANOVA (Session (1:9) x Group (iAG, cAG, ctrlAG) showed no significant Time main effect, Group main effect or Time x Group effect (Supplementary Table 6). The Phase (HDT, R) planned comparison was also not significant (Supplementary Table 7).

Concerning the Visual P3b amplitude, the rANOVA (Time (1:9) x Group (iAG, cAG, ctrlAG)) showed that the Time main effect, the Group main effect, and the Time x Group interaction were all non-significant (Supplementary Table 6). The HDT and R Phase comparison also showed no significant change for the Visual P3b amplitude (Supplementary Table 7).

A similar pattern was observed in the case of the Auditory P3b amplitude. The rANOVA (Time (1:9) x Group (iAG, cAG, ctrlAG)) (Supplementary Table 6) showed no significant Time main effect, Group main effect, and Time x Group interaction. The HDT and R Phase comparison was also not significant (Supplementary Table 7).

Descriptive statistics for the KSS are presented in Supplementary Table 8. A Time (1:9) x Group (iAG, cAG, ctrlAG) rANOVA was performed. Even though the Time main effect was significant, with increased KSS scores over Time, the Group main effect and the Time x Group interaction were not significant (Supplementary Table 9). In addition, no significant Phase difference was found between BDC and HDT or between BDC and R, however, a significant increment was detected in overall KSS scores between HDT and R (Supplementary Table 10).

Supplementary results regarding the effect of prolonged exposure to AG are also presented here. Planned comparison for Phase (HDT, R) x AG Groups (iAG, cAG) x Complexity (Simple, Complex) are reported for RT, Accuracy, and ERP components (P3a and P3b), for the HDTBR experimental groups (Supplementary Table 11) are detailed. No significant effect was found regarding RT, Accuracy, P3a, and P3b either in the Visual or the Auditory modality.

All relevant analyses for the HDTBR groups were also performed for the AMBCtrl group and are detailed in the following. Results include descriptive statistics for RT, Accuracy, ERP components P3a and P3b, and the KSS questionnaire, results for all performed rANOVAs, and planned comparisons for Phase effects. RT and Accuracy were analyzed using a Time (1:9) x Complexity (Simple, Complex) rANOVA in both modalities, while P3a and P3b amplitudes were analyzed with a Time (1:9) rANOVA. For the sake of compatibility with results concerning the HDTBR groups, the same set of planned contrasts was performed to compare sessions grouped into BDC, HDT, and R phases. The same set of contrasts was applied to all outcome variables. For Time comparison we kept the same phase names used in the HDTBR data (BDC, HDT, and R) for ease of understanding.

Supplementary results regarding the effect of Time on cognitive functioning on for RT, Accuracy, and the Karolinska Sleepiness Scale are also detailed in the following. Descriptive statistics for RT in the AMBCtrl group are presented in Supplementary Table 12.

Regarding Visual task performance in the AMBCtrl group, the rANOVA (Time (1:9) x Complexity (Simple, Complex)) showed a significant main effect of Time and Complexity, with higher RT in the Visual Complex Task compared to the Visual Simple Task (Supplementary Table 13). Running the same set of contrasts as on the HDTBR groups, a significant difference was found between the BDC and HDT with decreased RT during HDT compared to BDC, as well a significant interaction for Phase (BDC, HDT) x Complexity

(Simple, Complex) with the significant RT decrement between BDC to HDT to be more pronounced in the Visual Complex Task (Supplementary Table 14). RT showed no further significant decrement according to the HDT versus R Phase comparison and the Phase (HDT, R) x Complexity (Simple, Complex) interaction. Regarding the BDC versus R comparison, RT significantly decreased during R. The Phase (BDC, R) x Complexity (Simple, Complex) interaction was also significant, with the RT decrement being more pronounced in the Visual Complex Task compared to the Visual Simple Task during R.

As for the Auditory tasks, the rANOVA (Time (1:9) x Complexity (Simple, Complex)), revealed a significant Time and Complexity main effect, resulting in a higher RT in the Auditory Complex Task (Supplementary Table 13). However, the planned comparison showed no significant Phase effects (BDC vs HDT, BDC vs R, and HDT vs R) or Phase (BDC vs HDT, BDC vs R, and HDT vs R) x Complexity (Simple, Complex) interactions for Auditory Tasks (Supplementary Table 14).

Descriptive statistics for Accuracy in the AMBCtrl group are presented in Supplementary Table 15. Regarding the Visual Tasks, the rANOVA (Time (1:9) x Complexity (Simple, Complex)) revealed a non-significant Time main effect, while the Complexity did show a higher Accuracy (Supplementary Table 16). The planned comparison showed no significant Phase effects (BDC vs HDT, BDC vs R, and HDT vs R) or Phase (BDC vs HDT, BDC vs R, and HDT vs R) x Complexity (Simple, Complex) interactions (Supplementary Table 17).

The Auditory Tasks showed a similar pattern. The rANOVA (Time (1:9) x Complexity (Simple, Complex)) showed no significant Time main effect, while the Complexity main effect was significant with higher Accuracy in the Auditory Simple Task (Supplementary Table 16). The planned comparison showed no significant Phase effects (BDC vs HDT, BDC vs R, and

HDT vs R) or Phase (BDC vs HDT, BDC vs R, and HDT vs R) x Complexity (Simple, Complex) interactions (Supplementary Table 17).

Descriptive statistics for P3a and P3b amplitudes in the AMBCtrl group are presented in Supplementary Table 18. Concerning P3a, the rANOVA (Time (1:9)) showed no significant change in Visual P3a amplitude through the 9 sessions (Time main effect) (Supplementary Table 19). Using planned comparison none of the Phase effects (BDC to HDT, HDT to R, and BDC to R) were significant (Supplementary Table 20). There was also no significant Time main effect for Auditory P3a according to the rANOVA (Time (1:9)) (Supplementary Table 19). As for planned comparisons for Phase effects regarding Auditory P3a, there was a significant amplitude decrease from BDC to HDT, while the HDT vs R and the BDC and R Phase comparisons showed no significant Auditory P3a amplitude changes (Supplementary Table 20).

The P3b showed a similar pattern. The rANOVA (Time(1:9)) Time main effect was not significant for the Visual P3b amplitude (Supplementary Table 19). The planned comparisons also showed no significant Phase effects (BDC to HDT, HDT to R, and BDC to R) in the Visual modality (Supplementary Table 20). The rANOVA (Time (1:9)) also showed a not significant Time main effect for the Auditory P3b (Supplementary Table 19). As in the Visual modality, there was no significant change in Auditory P3b amplitude between BDC and HDT, HDT and R, or BDC and R (Supplementary Table 20).

Descriptive statistics for the KSS in the AMBCtrl group are presented in Supplementary Table 21. A Time (1:9) rANOVA was performed. In line with HDTBR data, the Time main effect was significant, with increased KSS scores over time (Supplementary Table 22). In addition, no significant Phase difference was found between BDC and HDT or between BDC and R,

however, a significant increment was detected in overall KSS scores between HDT and R (Supplementary Table 23).

Supplementary Tables

Supplementary Table 1. Descriptive statistics including means and standard deviations (SD) for RT in the HDTBR experimental groups. Results are presented based on task Modality (Visual, Auditory), and task Complexity (Simple, Complex) for all three HDTBR experimental groups (iAG, cAG and ctrlAG) and all three Phases (BDC, HDT, R).

Modality	Complexity	Group	BDC		HDT		R	
			Mean	SD	Mean	SD	Mean	SD
Visual	Simplex	iAG	293.944	38.424	295.636	33.214	323.128	90.776
		cAG	304.640	58.255	322.389	71.909	323.483	55.243
		ctrlAG	320.698	48.306	319.831	63.912	330.869	49.406
		Overall	306.35	50.26	313.06	60.19	325.72	67.95
	Complex	iAG	479.521	73.094	456.336	69.561	461.117	76.387
		cAG	476.855	66.231	468.339	66.962	467.526	72.338
		ctrlAG	487.243	63.722	456.785	76.167	469.171	74.857
		Overall	481.01	67.35	460.84	70.51	466.01	76.18
Auditory	Simplex	iAG	364.050	64.105	347.348	47.709	378.790	76.325
		cAG	353.276	57.509	376.570	81.966	376.414	73.174
		ctrlAG	383.124	65.268	382.958	83.633	389.335	77.942
		Overall	301.57	27.65	298.13	32.69	288.06	25.39
	Complex	iAG	506.446	70.535	485.228	66.135	498.084	62.557
		cAG	506.975	59.377	492.978	65.096	487.907	59.108
		ctrlAG	494.882	61.463	474.657	73.259	477.298	60.410
		Overall	469.55	59.87	455.78	67.39	451.11	77.53

Supplementary Table 2. Results regarding the Time (1:9) x Complexity (Simple, Complex) x Group (iAG, cAG, ctrlAG) rANOVA for RT and Accuracy in the HDTBR experimental groups. The two modalities (Visual and Auditory) were analyzed separately.

Measure	Modality	Effect	Df	F	p	Partial η^2
RT	Visual	Time(1:9)	8(152)	1.671	0.142	0.08
		Group(iAG, cAG, ctrlAG)	2(19)	0.128	0.880	0.01
		Complexity (Simple, Complex)	1(19)	234.16	<0.000	0.92
		Time x Group	16(152)	0.732	0.702	0.07
	Auditory	Time(1:9)	8(152)	1.123	0.351	0.05
		Group(iAG, cAG, ctrlAG)	2(19)	0.022	0.978	0.002
		Complexity (Simple, Complex)	1(19)	91.79	<0.000	0.82
		Time x Group	16(152)	0.943	0.517	0.09
Accuracy	Visual	Time(1:9)	8(152)	1.832	0.074	0.08
		Group(iAG, cAG, ctrlAG)	2(19)	0.35	0.711	0.03
		Complexity (Simple, Complex)	1(19)	6.79	0.017	0.26
		Time x Group	16(152)	1.110	0.367	0.10
	Auditory	Time(1:9)	8(152)	1.221	0.310	0.06
		Group(iAG, cAG, ctrlAG)	2(19)	1.40	0.271,	0.12
		Complexity (Simple, Complex)	1(19)	24.28	< 0.000	0.56
		Time x Group	16(152)	0.848	0.538	0.08

Supplementary Table 3. Results of the planned contrasts regarding RT and Accuracy for the Phase effect (HDT vs R) and the Phase (HDT vs R) x Complexity (Simple, Complex) interactions in the HDTBR experimental groups (iAG, cAG and ctrlAG averaged together). Results are presented based on task Modality (Visual, Auditory), and task Complexity (Simple, Complex).

Measure	Modality	Effect	Df	F	p	Partial η^2
RT	Visual	HDT, R	1(19)	1.538	0.229	0.075
		HDT, R × Simple, Complex	1(19)	0.932	0.346	0.047
	Auditory	HDT, R	1(19)	1.423	0.247	0.070
		HDT, R × Simple, Complex	1(19)	1.082	0.311	0.054
Accuracy	Visual	HDT, R	1(19)	1.264	0.274	0.062
		HDT, R × Simple, Complex	1(19)	0.128	0.723	0.007
	Auditory	HDT, R	1(19)	3.467	0.078	0.154
		HDT, R × Simple, Complex	1(19)	0.010	0.920	0.001

Supplementary Table 4. Descriptive statistics including means and standard deviations (SD) for Accuracy in the HDTBR experimental groups. Results are presented based on task Modality (Visual, Auditory), and task Complexity (Simple, Complex) for all three HDTBR experimental groups (iAG, cAG and ctrlAG) and all three Phases (BDC, HDT, R).

Modality	Complexity	Group	BDC		HDT		R	
			Mean	SD	Mean	SD	Mean	SD
Visual	Simplex	iAG	0.979	0.045	0.985	0.033	0.969	0.061
		cAG	0.965	0.073	0.973	0.077	0.962	0.068
		ctrlAG	0.969	0.061	0.968	0.081	0.979	0.042
		Overall	0.97	0.06	0.98	0.07	0.97	0.06
	Complex	iAG	0.942	0.073	0.949	0.060	0.953	0.065
		cAG	0.953	0.049	0.965	0.051	0.958	0.054
		ctrlAG	0.959	0.042	0.964	0.053	0.956	0.067
		Overall	0.95	0.05	0.96	0.05	0.96	0.06
Auditory	Simplex	iAG	0.988	0.027	0.994	0.019	0.986	0.029
		cAG	0.986	0.027	0.985	0.030	0.979	0.039
		ctrlAG	0.987	0.029	0.989	0.026	0.986	0.032
		Overall	0.99	0.03	0.99	0.02	0.98	0.033
	Complex	iAG	0.894	0.138	0.909	0.119	0.911	0.124
		cAG	0.951	0.058	0.966	0.046	0.952	0.058
		ctrlAG	0.938	0.085	0.962	0.097	0.955	0.123
		Overall	0.96	0.05	0.96	0.06	0.96	0.06

Supplementary Table 5. Descriptive statistics including means and standard deviations (SD) for P3a and P3b amplitude in the HDTBR experimental groups. Results are presented based on task Modality (Visual, Auditory) for all three HDTBR experimental groups (iAG, cAG and ctrlAG) and all three Phases (BDC, HDT, R).

Modality	Component	Group	BDC		HDT		R	
			Mean	SD	Mean	SD	Mean	SD
Visual	P3a	iAG	2.472	3.319	2.434	3.148	1.374	3.958
		cAG	1.970	2.474	1.198	2.225	0.909	2.209
		ctrlAG	3.471	3.296	3.033	3.080	3.039	3.498
		Overall	2.607	3.025	2.175	2.865	1.734	3.340
	P3b	iAG	4.300	1.166	3.734	1.595	3.962	1.407
		cAG	4.083	1.369	3.101	1.578	3.444	1.497
		ctrlAG	3.823	2.365	3.402	2.056	3.538	2.305
		Overall	4.069	1.671	3.398	1.728	3.639	1.757
Auditory	P3a	iAG	6.294	1.134	5.915	1.325	6.722	2.016
		cAG	5.374	2.678	4.667	1.840	5.141	2.978
		ctrlAG	6.833	2.354	6.442	2.389	7.311	2.887
		Overall	6.131	2.224	5.629	2.006	6.334	2.799
	P3b	iAG	2.829	1.494	2.295	1.565	2.383	1.441
		cAG	3.538	2.088	2.996	1.989	3.329	1.945
		ctrlAG	3.292	1.492	3.247	1.234	3.107	1.725
		Overall	3.234	1.720	2.853	1.655	2.957	1.749

Supplementary Table 6. Results regarding the Time (1:9) x Group (iAG, cAG, ctrlAG) rANOVA for P3a and P3b amplitudes in the HDTBR experimental groups. The two modalities (Visual and Auditory) were analyzed separately.

Measure	Modality	Effect	Df	F	p	Partial η^2
P3a	Visual	Time(1:9)	8(152)	2.368	0.040	0.11
		Group(iAG, cAG, ctrlAG)	2(19)	0.832	0.450	0.08
		Time x Group	16(152)	0.475	0.955	0.04
	Auditory	Time(1:9)	8(152)	1.097	0.366	0.05
		Group(iAG, cAG, ctrlAG)	2(19)	1.970	0.16	0.17
		Time x Group	16(152)	0.653	0.834	0.06
P3b	Visual	Time(1:9)	8(152)	1.854	0.072	0.089
		Group(iAG, cAG, ctrlAG)	2(19)	0.212	0.811	0.02
		Time x Group	16(152)	0.602	0.878	0.060
	Auditory	Time(1:9)	8(152)	0.893	0.522	0.045
		Group(iAG, cAG, ctrlAG)	2(19)	0.640	0.651	0.063
		Time x Group	16(152)	0.829	0.648	0.08

Supplementary Table 7. Results of the planned contrasts regarding P3a and P3b amplitudes for the Phase effect (HDT vs R) in the HDTBR experimental groups (iAG, cAG and ctrlAG averaged together). Results are presented based on task Modality (Visual, Auditory).

Measure	Modality	Effect	Df	F	p	Partial η^2
P3a	Visual	HDT, R	1(19)	1.566	0.225	0.076
	Auditory	HDT, R	1(19)	3.900	0.062	0.170
P3b	Visual	HDT, R	1(19)	1.342	0.261	0.066
	Auditory	HDT, R	1(19)	0.210	0.653	0.011

Supplementary Table 8. Descriptive statistics including means and standard deviations (SD) for the Karolinska Sleepiness Scale (KSS). Results are presented for the three HDTBR experimental groups averaged together (iAG, cAG and ctrlAG) for all three Phases (BDC, HDT, R).

Measure	Group	BDC		HDT		R	
		Mean	SD	Mean	SD	Mean	SD
KSS	HDTBR	4.614	1.807	4.273	1.436	5.136	1.788

Supplementary Table 9. Results regarding the Time (1:9) x Group (iAG, cAG, ctrlAG) rANOVA for KSS in the HDTBR experimental groups.

Measure	Effect	Df	F	p	Partial η^2
KSS	Time(1:9)	8(152)	2.420	0.017	0.113
	Group(iAG, cAG, ctrlAG)	2(19)	0.052	0.950	0.005
	Time x Group	16(152)	1.268	0.225	0.118

Supplementary Table 10. Results of the planned contrasts for KSS regarding Phase effects (BDC vs HDT, BDC vs R, and HDT vs R) in the HDTBR experimental groups (iAG, cAG and ctrlAG averaged together).

Measure	Effect	Df	F	p	Partial η^2
KSS	BDC, HDT	1(19)	3.708	0.069	0.163
	BDC, R	1(19)	3.007	0.099	0.137
	HDT, R	1(19)	10.308	0.005	0.352

Supplementary Table 11. Results of the planned contrasts regarding RT, Accuracy, P3a, and P3b amplitudes or Phase (HDT, R) x AG Groups (iAG, cAG) x Complexity (Simple, Complex) in the two HDTBR experimental groups receiving AG (iAG, cAG). Results are presented based on task Modality (Visual, Auditory).

Measure	Modality	Effect	Df	F	p	Partial η^2
RT	Visual	HDT, R × iAG, cAG × Simple, Complex	1(19)	1.143	0.298	0.057
	Auditory	HDT, R × iAG, cAG × Simple, Complex	1(19)	0.418	0.526	0.022
Accuracy	Visual	HDT, R × iAG, cAG × Simple, Complex	1(19)	1.817	0.194	0.087
	Auditory	HDT, R × iAG, cAG × Simple, Complex	1(19)	1.755	0.201	0.085
P3a	Visual	HDT, R × iAG, cAG	1(19)	0.789	0.386	0.040
	Auditory	HDT, R × iAG, cAG	1(19)	0.143	0.709	0.007
P3b	Visual	HDT, R × iAG, cAG	1(19)	0.055	0.818	0.003
	Auditory	HDT, R × iAG, cAG	1(19)	0.243	0.628	0.013

Supplementary Table 12. Descriptive statistics including means and standard deviations (SD) for RT in the AMBCtrl group. Results are presented based on task Modality (Visual, Auditory), and task Complexity (Simple, Complex) for the AMBCtrl group in all three Phases (BDC, HDT, R).

Modality	Complexity	BDC		HDT		R	
		Mean	SD	Mean	SD	Mean	SD
Visual	Simple	301.57	27.65	298.13	32.69	288.06	25.39
	Complex	472.79	55.35	442.70	58.20	434.64	63.24
Auditory	Simplex	365.71	48.77	355.13	46.76	347.86	46.48
	Complex	469.55	59.87	455.78	67.39	451.11	77.53

Supplementary Table 13. Results regarding the Time (1:9) x Complexity (Simple, Complex) rANOVA for RT in the AMBCtrl group. The two modalities (Visual and Auditory) were analyzed separately.

Measure	Modality	Effect	Df	F	p	Partial η^2
RT	Visual	Time(1:9)	8(64)	7.193	<0.000	0.47
		Complexity (Simple, Complex)	1(8)	96.023	< 0.000	0.92
	Auditory	Time(1:9)	8(64)	4.088	<0.000	0.33
		Complexity (Simple, Complex)	1(8)	58.741	< 0.000	0.88

Supplementary Table 14. Results of the planned contrasts regarding RT for the Phase effects (BDC vs HDT, BDC vs R, and HDT vs R) and the Phase (BDC vs HDT, BDC vs R, and HDT vs R) x Complexity (Simple, Complex) interactions in the AMBCtrl group. Results are presented based on task Modality (Visual, Auditory), and task Complexity (Simple, Complex).

Measure	Modality	Effect	Df	F	p	Partial η^2
RT	Visual	BDC, HDT	1(8)	11.658	0.009	0.593
		BDC, HDT × Simple, Complex	1(8)	17.780	0.002	0.690
		HDT, R	1(8)	3.173	0.112	0.284
		HDT, R × Simple, Complex	1(8)	0.584	0.466	0.068
		BDC, R	1(8)	8.396	0.019	0.512
		BDC, R × Simple, Complex	1(8)	9.839	0.013	0.552
	Auditory	BDC, HDT	1(8)	1.930	0.202	0.194
		BDC, HDT × Simple, Complex	1(8)	0.908	0.368	0.102
		HDT, R	1(8)	1.352	0.278	0.145
		HDT, R × Simple, Complex	1(8)	0.885	0.374	0.100
		BDC, R	1(8)	2.667	0.141	0.250
		BDC, R × Simple, Complex	1(8)	0.002	0.958	0.000

Supplementary Table 15. Descriptive statistics including means and standard deviations (SD) for Accuracy (ACC) in the AMBCtrl group. Results are presented based on task Modality (Visual, Auditory), and task Complexity (Simple, Complex) for the AMBCtrl group in all three Phases (BDC, HDT, R).

Modality	Complexity	BDC		HDT		R	
		Mean	SD	Mean	SD	Mean	SD
Visual	Simple	0.98	0.04	0.98	0.05	0.96	0.07
	Complex	0.96	0.04	0.96	0.05	0.96	0.06
Auditory	Simplex	0.99	0.05	0.99	0.03	0.99	0.03
	Complex	0.96	0.05	0.96	0.06	0.96	0.06

Supplementary Table 16. Results regarding the Time (1:9) x Complexity (Simple, Complex) rANOVA for Accuracy in the AMBCtrl group. The two modalities (Visual and Auditory) were analyzed separately.

Measure	Modality	Effect	Df	F	p	Partial η^2
Accuracy	Visual	Time(1:9)	8(64)	1.110	0.368	0.12
		Complexity (Simple, Complex)	1(8)	6.55	0.033	0.45
	Auditory	Time(1:9)	8(64)	0.517	0.838	0.06
		Complexity (Simple, Complex)	1(8)	17.29	0.003	0.68

Supplementary Table 17. Results of the planned contrasts regarding Accuracy for the Phase effects (BDC vs HDT, BDC vs R, and HDT vs R) and the Phase (BDC vs HDT, BDC vs R, and HDT vs R) x Complexity (Simple, Complex) interactions in the AMBCtrl group. Results are presented based on task Modality (Visual, Auditory), and task Complexity (Simple, Complex).

Measure	Modality	Effect	Df	F	p	Partial η^2
Accuracy	Visual	BDC, HDT	1(8)	1.268	0.292	0.137
		BDC, HDT × Simple, Complex	1(8)	3.217	0.110	0.287
		HDT, R	1(8)	0.169	0.691	0.021
		HDT, R × Simple, Complex	1(8)	0.434	0.528	0.051
		BDC, R	1(8)	0.865	0.379	0.098
		BDC, R × Simple, Complex	1(8)	1.404	0.270	0.149
	Auditory	BDC, HDT	1(8)	0.001	0.973	0.000
		BDC, HDT × Simple, Complex	1(8)	0.010	0.922	0.001
		HDT, R	1(8)	0.014	0.906	0.002
		HDT, R × Simple, Complex	1(8)	0.0006	0.980	0.000
		BDC, R	1(8)	0.007	0.934	0.001
		BDC, R × Simple, Complex	1(8)	0.007	0.933	0.001

Supplementary Table 18. Descriptive statistics including means and standard deviations (SD) for P3a and P3b amplitudes in the AMBCtrl group. Results are presented based on task Modality (Visual, Auditory) in all three Phases (BDC, HDT, R).

Modality	Components	BDC		HDT		R	
		Mean	SD	Mean	SD	Mean	SD
Visual	P3a	5.438	2.103	5.226	3.072	4.633	3.448
	P3b	4.185	2.574	3.889	2.605	3.404	2.156
Auditory	P3a	7.017	1.882	7.038	2.355	7.649	1.980
	P3b	3.953	2.059	3.379	1.889	3.162	2.101

Supplementary Table 19. Results regarding the Time (1:9) rANOVA for P3a and P3b amplitudes in the AMBCtrl group. The two modalities (Visual and Auditory) were analyzed separately.

Measure	Modality	Effect	Df	F	p	Partial η^2
P3a	Visual	Time(1:9)	8(64)	0.982	0.451	0.10
	Auditory	Time(1:9)	8(64)	1.012	0.436	0.11
P3b	Visual	Time(1:9)	8(64)	1.049	0.403	0.41
	Auditory	Time(1:9)	8(64)	1.392	0.268	0.217

Supplementary Table 20. Results of the planned contrasts regarding P3a and P3b amplitudes for the Phase effect (HDT vs R) in the AMBCtrl group. Results are presented based on task Modality (Visual, Auditory).

Measure	Modality	Effect	Df	F	p	Partial η^2
P3a	Visual	BDC, HDT	1(8)	0.079	0.784	0.010
		HDT, R	1(8)	1.309	0.285	0.141
		BDC, R	1(8)	1.836	0.191	0.187
	Auditory	BDC, HDT	1(8)	5.701	0.044	0.416
		HDT, R	1(8)	2.199	0.176	0.216
		BDC, R	1(8)	0.321	0.577	0.039
P3b	Visual	BDC, HDT	1(8)	0.305	0.596	0.037
		HDT, R	1(8)	2.993	0.122	0.272
		BDC, R	1(8)	1.586	0.243	0.165
	Auditory	BDC, HDT	1(8)	4.239	0.073	0.346
		HDT, R	1(8)	0.387	0.551	0.046
		BDC, R	1(8)	3.53	0.097	0.306

Supplementary Table 21. Descriptive statistics including means and standard deviations (SD) for the Karolinska Sleepiness Scale. Results are presented for the AMBCtrl group in all three Phases (BDC, HDT, R).

Measure	Group	BDC		HDT		R	
		Mean	SD	Mean	SD	Mean	SD
KSS	AMBCtrl	3.15	1.089	3.375	0.838	3.867	1.196

Supplementary Table 22. Results regarding the Time (1:9) for KSS in the AMBCtrl experimental group.

Measure	Effect	Df	F	p	Partial η^2
KSS	Time(1:9)	8(72)	2.896	0.007	0.243

Supplementary Table 23. Results of the planned contrasts for KSS regarding Phase effects (BDC vs HDT, BDC vs R, and HDT vs R) in the AMBCtrl experimental group.

Measure	Effect	Df	F	p	Partial η^2
KSS	BDC, HDT	1(9)	0.167	0.693	0.009
	BDC, R	1(9)	4.922	0.054	0.206
	HDT, R	1(9)	7.508	0.023	0.283
