# Supplementary material for: Effect of artificial gravity on neurocognitive performance during head-down tilt bedrest
Source: NPJ Microgravity. 2024 Jun 5;10:59. doi: 10.1038/s41526-024-00405-4 (PMC11153507; doi:10.1038/s41526-024-00405-4)
Supplement: Supplementary file 2 — Reporting Summary [file 41526_2024_405_MOESM2_ESM.pdf]

nature portfolio

Corresponding author(s): Anna Altbäcker

Last updated by author(s): May 8, 2024

Reporting Summary

Nature Portfolio wishes to improve the reproducibility of the work that we publish. This form provides structure for consistency and transparency in reporting. For further information on Nature Portfolio policies, see our Editorial Policies and the Editorial Policy Checklist.

Statistics

For all statistical analyses, confirm that the following items are present in the figure legend, table legend, main text, or Methods section.

n/a	Confirmed	
☐	☒	The exact sample size (*n*) for each experimental group/condition, given as a discrete number and unit of measurement
☐	☒	A statement on whether measurements were taken from distinct samples or whether the same sample was measured repeatedly
☐	☒	The statistical test(s) used AND whether they are one- or two-sided<br>*Only common tests should be described solely by name; describe more complex techniques in the Methods section.*
☐	☒	A description of all covariates tested
☐	☒	A description of any assumptions or corrections, such as tests of normality and adjustment for multiple comparisons
☐	☒	A full description of the statistical parameters including central tendency (e.g. means) or other basic estimates (e.g. regression coefficient) AND variation (e.g. standard deviation) or associated estimates of uncertainty (e.g. confidence intervals)
☐	☒	For null hypothesis testing, the test statistic (e.g. *F*, *t*, *r*) with confidence intervals, effect sizes, degrees of freedom and *P* value noted<br>*Give P values as exact values whenever suitable.*
☒	☐	For Bayesian analysis, information on the choice of priors and Markov chain Monte Carlo settings
☒	☐	For hierarchical and complex designs, identification of the appropriate level for tests and full reporting of outcomes
☐	☒	Estimates of effect sizes (e.g. Cohen's *d*, Pearson's *r*), indicating how they were calculated

Our web collection on statistics for biologists contains articles on many of the points above.

Software and code

Policy information about availability of computer code

Data collection	EEGLAB toolbox, Matlab.
Data analysis	Statistica 13.5

For manuscripts utilizing custom algorithms or software that are central to the research but not yet described in published literature, software must be made available to editors and reviewers. We strongly encourage code deposition in a community repository (e.g. GitHub). See the Nature Portfolio guidelines for submitting code & software for further information.

Data

Policy information about availability of data

All manuscripts must include a data availability statement. This statement should provide the following information, where applicable:

- Accession codes, unique identifiers, or web links for publicly available datasets
- A description of any restrictions on data availability
- For clinical datasets or third party data, please ensure that the statement adheres to our policy

Data analysis scripts and data underlying statistical analyses are available at:
https://figshare.com/projects/NeuroGravity/161770

Research involving human participants, their data, or biological material

Policy information about studies with human participants or human data. See also policy information about sex, gender (identity/presentation), and sexual orientation and race, ethnicity and racism.

Reporting on sex and gender	Gender was assessed using self-reporting methods. The final sample comprised seven participant in the iAG, (5 males, 2 females), eight in the cAG (5 males, 3 females), and seven in ctrlAG, (5 males, 2 females), and nine participants in the ambulatory control group (5 males, 4 female). The sample size did not enable well-powered statistical analyses about gender differences, which was included as a limitation of the study.
Reporting on race, ethnicity, or other socially relevant groupings	Race, ethnicity, or Socially relevant categorization variables were not used in this study.
Population characteristics	Healthy adults.
Recruitment	The study enrolled healthy individuals (16 men, 8 women), who had been submitted to detailed medical and psychological screening. We are not aware of any bias towards selection.
Ethics oversight	The AGBRESA project was approved by the Ethics committee of the Northern Rhine Medical Association (Ärztekammer Nordrhein, application No. 2018143) in Düsseldorf, Germany, as well as the Federal Office for Radiation Protection (Bundesamt für Strahlenschutz, application No. 22464/2018-074-R-G), while the ethical approval for the AMBCtrl was given by the Ethics committee of the German Sport University Cologne (No. 173/2018). The study was registered at the German Clinical Trials Register (DRKS00015677).

Note that full information on the approval of the study protocol must also be provided in the manuscript.

Field-specific reporting

Please select the one below that is the best fit for your research. If you are not sure, read the appropriate sections before making your selection.

☒ Life sciences ☐ Behavioural & social sciences ☐ Ecological, evolutionary & environmental sciences

For a reference copy of the document with all sections, see nature.com/documents/nr-reporting-summary-flat.pdf

Life sciences study design

All studies must disclose on these points even when the disclosure is negative.

Sample size	This study was part of the Artificial Gravity Bed Rest - European Space Agency (AGBRESA) project, with pre-defined sample size based on their sample size calculation. Therefore, no sample size calculation was conducted as part of this study.
Data exclusions	Out of twenty-four subjects, two participants were excluded from the analysis, one from the iAG group due to low behavioral performance (in both Complex tasks Accuracy was worse than 60% during at least 3 sessions) and one from the ctrlAG group due to low EEG quality (number of sweeps was below 20 in each condition during at least an entire session). These were pre-established criteria.
Replication	The study was meticulously designed, with careful selection of appropriate statistical analyses, and results were reported objectively, effect sizes were also reported. Transparency was ensured by providing clear descriptions of the study design, materials, data collection, and data analysis. Additionally, link for data analysis scripts and data underlying statistical analyses was provided and all additional analyses were included as supplementary material.
Randomization	Participants were randomly assigned to the experimental groups: intermittent AG, continuous AG, and HDTBR control group without AG exposure, and ambulatory control group.
Blinding	Investigators and participants were not blinded to group allocation, as the distinctive characteristics of the study design made the protocol easily recognizable within each group.

Reporting for specific materials, systems and methods

We require information from authors about some types of materials, experimental systems and methods used in many studies. Here, indicate whether each material, system or method listed is relevant to your study. If you are not sure if a list item applies to your research, read the appropriate section before selecting a response.

Materials & experimental systems

n/a	Involved in the study
☒	☐ Antibodies
☒	☐ Eukaryotic cell lines
☒	☐ Palaeontology and archaeology
☒	☐ Animals and other organisms
☒	☐ Clinical data
☒	☐ Dual use research of concern
☒	☐ Plants

Methods

n/a	Involved in the study
☒	☐ ChIP-seq
☒	☐ Flow cytometry
☒	☐ MRI-based neuroimaging

Plants

Seed stocks	*Report on the source of all seed stocks or other plant material used. If applicable, state the seed stock centre and catalogue number. If plant specimens were collected from the field, describe the collection location, date and sampling procedures.*
Novel plant genotypes	*Describe the methods by which all novel plant genotypes were produced. This includes those generated by transgenic approaches, gene editing, chemical/radiation-based mutagenesis and hybridization. For transgenic lines, describe the transformation method, the number of independent lines analyzed and the generation upon which experiments were performed. For gene-edited lines, describe the editor used, the endogenous sequence targeted for editing, the targeting guide RNA sequence (if applicable) and how the editor was applied.*
Authentication	*Describe any authentication procedures for each seed stock used or novel genotype generated. Describe any experiments used to assess the effect of a mutation and, where applicable, how potential secondary effects (e.g. second site T-DNA insertions, mosiacism, off-target gene editing) were examined.*
